# Sex differences in the impact of resistance exercise load on muscle damage: A protocol for a randomised parallel group trial

**DOI:** 10.1371/journal.pone.0275221

**Published:** 2022-09-29

**Authors:** Alice G. Pearson, Lindsay S. Macnaughton, Karen Hind

**Affiliations:** Department of Sport and Exercise Sciences, Durham University, Durham, United Kingdom; Prince Sattam Bin Abdulaziz University, College of Applied Medical Sciences, SAUDI ARABIA

## Abstract

**Introduction:**

Resistance training can induce skeletal muscle hypertrophy and strength gains, but is also associated with acute muscle damage, characterised by muscle soreness, impaired muscle function, and structural damage to muscle cell membranes and its components. These consequences can be detrimental to future exercise performance and dampen long-term training adaptations. Previous research has considered resistance exercise intensity as a factor in exercise-induced muscle damage (EIMD), though a clear direction of the findings has not yet been established. Further, female populations are heavily underrepresented in this field of study. Therefore, we here propose a study protocol designed to examine sex differences in the muscle damage response to resistance exercise performed with low or high loads in a population of untrained, young adults.

**Methods:**

This study will employ a randomised parallel group design. Twenty-four males and 24 females will perform an acute leg-based resistance exercise session at either 30% (low-load) or 80% (high-load) of their pre-determined one-repetition maximum (1RM). Maximal leg strength will be determined by a 1RM test 3 wk before and 72 and 168 h after the exercise bout. Additionally, muscle damage will be assessed immediately before the exercise bout and immediately, 24, 48, 72, and 168 h after the exercise bout through measures of muscle soreness, limb circumference, range of motion, and serum concentrations of creatine kinase and interleukin-6. The outcomes of this trial could inform sex-specific resistance training recommendations and help bridge the sex data gap in sport and exercise science research.

## Introduction

Individuals engage in resistance exercise for a multitude of reasons, not limited to improving sport performance, enhancing body composition, delaying sarcopenia, and maintaining general health and psychological well-being. At a rudimentary level, repeated bouts of resistance exercise may culminate in skeletal muscle hypertrophy and increases in muscle force-generating capacity [1]. Research over the past decade has challenged the notion that traditional high-load [>70% one repetition maximum (1RM)] resistance training is necessary to induce skeletal muscle adaptations [2]. Schoenfeld and colleagues suggest that training with exercise loads as low as 30% 1RM elicits comparable skeletal muscle hypertrophy and isometric strength gains to with high loads [2]. Nevertheless, the capacity of skeletal muscle to adapt to resistance training may be limited by acute muscle fibre damage [3].

Skeletal muscle damage is a potential consequence of unaccustomed or eccentrically-biased muscle contractions [4]. At a cellular level, the effects of the damage are targeted toward the structural components of sarcomeres and to the sarcolemma, resulting in the leakage of intramuscular proteins (e.g., creatine kinase and myoglobin) into systemic circulation [5–7]. However, of greatest deterrent to the exercising individual are those events that present themselves on the surface. Resistance exercise-induced muscle damage (EIMD) may cause muscle soreness, limb swelling, reduced flexibility, and most notably, decreased muscle force-generating capacity [8–10]. The presence of these impairments for up to a week following EIMD [11] increases the required recovery time preceding subsequent exercise bouts. In the absence of sufficient recovery, exercise performance may suffer and, by this means, restrict the occurrence of maximal training adaptations. Therefore, gaining understanding of the mechanisms underpinning EIMD and developing mitigation strategies is paramount to researchers and exercising individuals alike.

The severity of EIMD may be predetermined by several extrinsic variables of the exercise bout. These include the muscle group exercised [12], total number of eccentric contractions, velocity of movement [13, 14], muscle length at which the contraction is initiated and maximum force is generated [15, 16], and the maximum force produced [17]. However, due to the inherent relationship between muscle force and length (i.e., length-tension curve [18]), it is challenging to ascertain whether the severity of EIMD is determined by the force production or length of the muscle, as they characteristically peak in unison. Therefore, it is important to understand the effects of these variables in isolation on EIMD. Studies in animals [19, 20] and humans [15, 16, 21–23] have consistently demonstrated more severe EIMD when the targeted muscle is stretched over a greater range of muscle lengths. Conversely, when the maximum force output of the muscle is manipulated (i.e., by varying the exercise load or electrostimulation intensity) the impact on EIMD magnitude is less transparent, at least in humans. For example, of the studies comparing the impact of resistance exercise performed with low and high loads on various markers of muscle damage, several reported no differences between exercise loads for maximal voluntary contraction [24–26], subjective muscle soreness [24, 27–30], or creatine kinase activity [25, 27–32]. These data suggest that the muscle force generated during contraction is not necessarily a prerequisite to EIMD. Although, in the instances whereby EIMD differed between exercise loads, the outcome measures predominantly favoured the low-load condition [17, 33–35]. In this regard, performing resistance exercise with lighter loads may be a viable alternative to traditional high-load resistance exercise to attenuate EIMD, without dampening muscular adaptations to training.

The current literature surrounding the EIMD response to varied exercise loads has underrepresented exclusively female populations. Orssatto and colleagues report combined data from older adult males and females, in which resistance exercise performed at 85% relative to 60% 1RM significantly reduced maximal isometric torque of the knee extensor and flexor muscles [35]. Young females alone were studied by Alvarez and colleagues, yet the exercise protocol incorporated blood flow restriction in the low-load condition only, which was associated with mild muscle soreness [36]. Therefore, the impact of exercise load on EIMD is poorly understood in females.

The question as to whether EIMD is sex-specific is heavily debated among researchers [37–39]. Fundamental physiological differences between males and females, including but not limited to skeletal muscle fibre type distribution, body adiposity, bone mineral density, maximal strength, muscle fatigue resistance, and sex hormone milieu [40–46] may cause disparate EIMD responses between sexes. While early animal models strongly support a role of oestrogens in protecting against EIDM [47–52], this argument does not hold in humans. Following eccentric exercise, females have experienced more severe strength impairments [53, 54], inflammation [55], and intramuscular protein leakage [56] relative to their male counterparts. Consistent with previous animal models, some studies have reported attenuated EIMD in females [57–59], whereas others have failed to identify sex differences [55, 60–65]. Furthermore, the findings from studies investigating the effects of hormone replacement therapy [66], hormonal contraceptive use [54, 67], and menstrual cycle phase [68, 69] are ambivalent. The determination of sex-dependent responses to EIMD is warranted to better understand the factors influencing and potential management strategies for EIMD.

The following protocol is designed to address two aims. First, to investigate the acute muscle damage response to a single bout of resistance exercise performed with low or high loads. Second, to compare the EIMD response between untrained male and female young adults. Combined with the growing body of literature, the outcomes would inform the development of sex-specific training recommendations to maximise adaptations while minimising muscle damage. This protocol may be replicated or used as a basis for future research on sex differences in EIMD.

## Methods and analysis

### Study design

**Fig 1:**
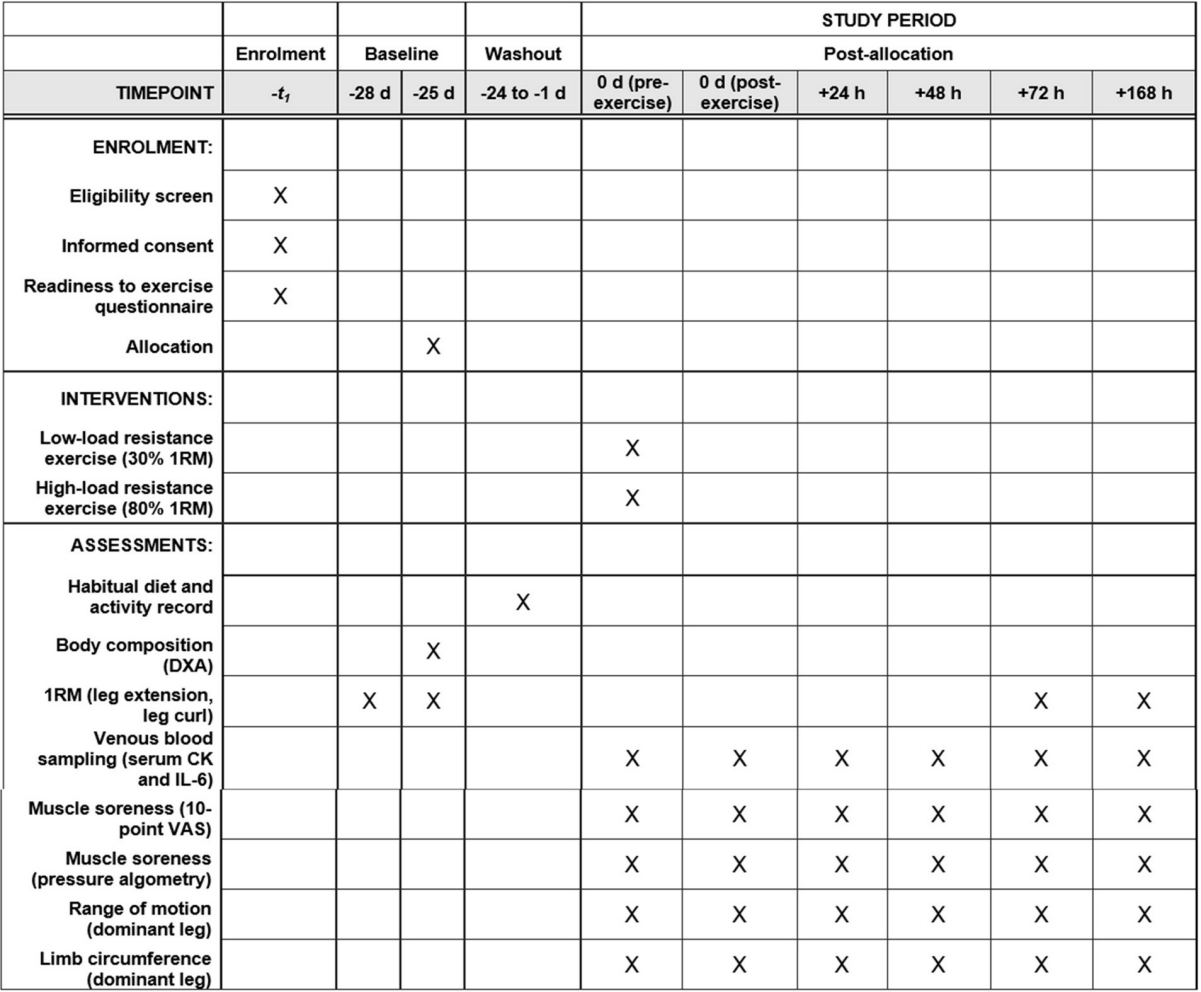
SPIRIT Schedule of enrolment, interventions, and assessments. 1RM = one repetition maximum; DXA = dual-energy X-ray absorptiometry; CK = creatine kinase; IL-6 = interleukin-6; VAS = visual analogue scale.

**Fig 2:**
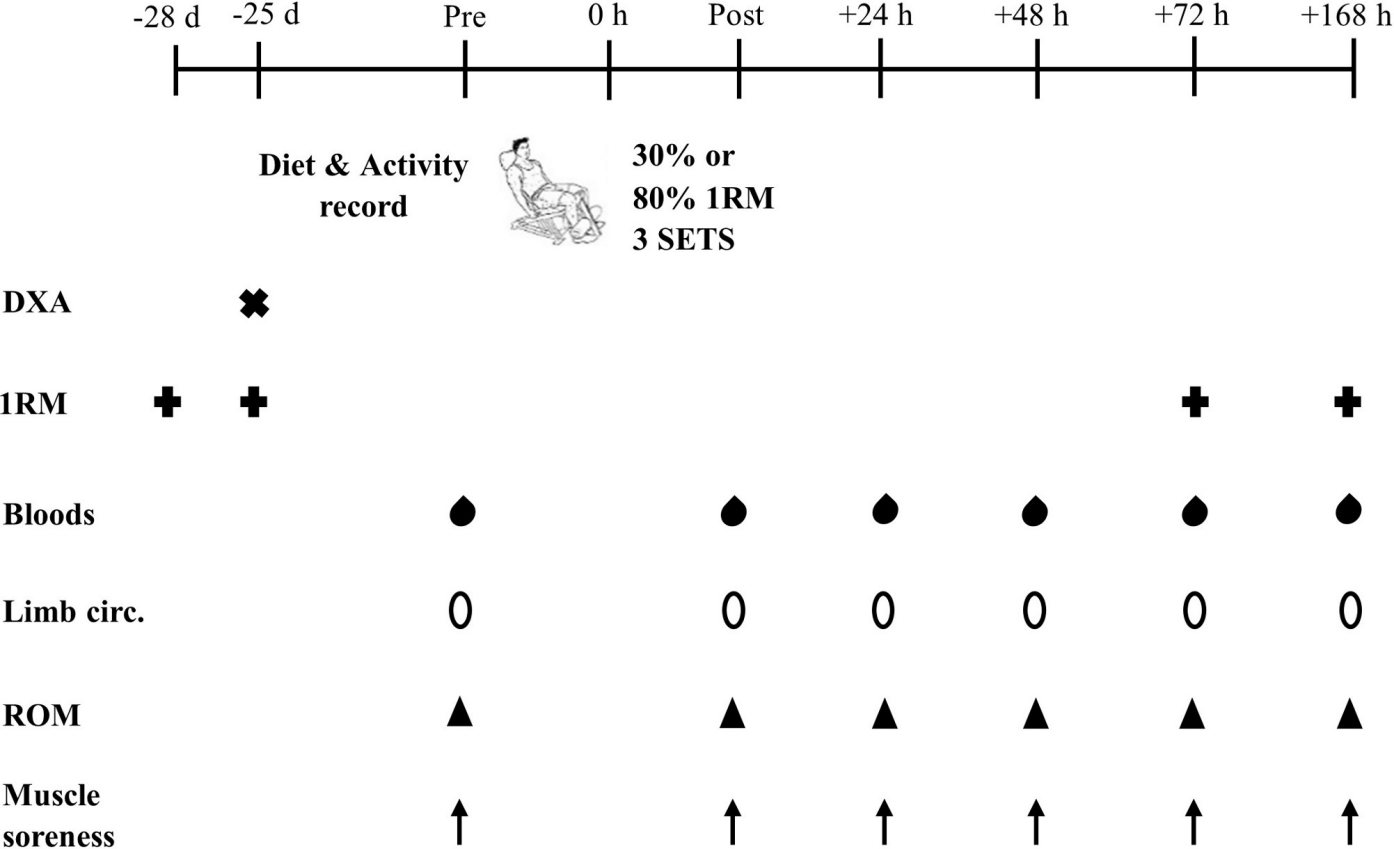
Schematic overview of study design. Note: DXA = dual-energy X-ray absorptiometry; 1RM = one repetition maximum; limb circ. = limb circumference; ROM = range of movement.

A randomised, parallel group design will be employed to examine sex differences in the response to low-load and high-load resistance exercise for indirect markers of muscle damage. Following an initial virtual screening to assess participant eligibility, the first two laboratory visits will act as familiarisation sessions, during which maximal leg strength and body composition will be assessed. Participants will then be stratified by sex and randomised to either a low-load (30% 1RM) or high-load (80% 1RM) exercise condition. A three-week period will separate the familiarisation visits and the muscle damage exercise protocol to reduce the influence of repeated-bout effects and to allow all measures to be conducted during the late follicular phase of the menstrual cycle in female participants. Venous blood samples and measures of limb circumference, range of motion, and muscle soreness will be obtained before, immediately after, and 24, 48, 72, and 168 h after the exercise bout. These time points should allow the peak values for each marker to be captured, which are expected to progressively return to baseline values by 168 h post-exercise. Additionally, during the 72 and 168 h post-exercise visits, the assessment of maximal leg strength will be repeated. Maximal leg strength will not be assessed immediately, 24, and 48 h post-exercise to mimic a realistic training schedule in which a muscle group would be rested for ≥2 d. A SPIRIT schedule of enrolment, interventions, and assessments is presented in *Fig 1* and a schematic overview of the study design is presented in *Fig 2*.

### Participants and recruitment

48 healthy participants (24 males and 24 females, aged 18–35 years) will be recruited to participate in this study. Participants will not have performed regular (≥twice weekly) resistance or eccentrically-biased exercise during the previous six-month period. Participants will be recruited primarily from the Durham University student and staff cohort using a convenience sampling strategy with sex stratification. Prospective participants will be invited to volunteer for the study via email and social media (Twitter, LinkedIn) advertisements, as well as word-of-mouth. A statistical power analysis was conducted using G*Power 3.1 to determine the study sample size. The power calculation was based on a similar study by Orssatto et al. [35] which reported a significant difference in the change in isometric MVC from baseline to 24 h post-exercise between low-load and high-load groups of 6.5%. The post-exercise change in MVC is deemed the most valid indirect indicator of EIMD (Damas et al., 2016). The calculation revealed that 12 participants per group (48 total) were required to have 80% power to detect a significant difference between groups when using a dependent *t* test with a 0.05 two-sided significance level. Therefore, a total of 56 participants (14 low-load male, 14 low-load female, 14 high-load male, 14 high-load female) will be recruited to allow for 20% drop-out.

### Inclusion criteria

The study will include male and female adults who:

1. Are aged 18–35 years old
2. Are a healthy weight (BMI >18.5 and <25 kg/m^2^)
3. Have no recent (previous 6 months) experience in resistance exercise
4. Are able to perform leg-based resistance exercise
5. Are free from musculoskeletal injury (past 6 months)
6. Are eumenorrheic (females only; regular menstrual cycle over the past 12 months)

### Exclusion criteria

The study will not include participants who:

1. Habitually perform resistance exercise or eccentrically-biased exercise (e.g., downhill running)
2. Habitually (i.e., twice per week for previous 1-month period) consume ergogenic aids (e.g., creatine monohydrate), protein-based supplements, non-steroidal anti-inflammatory drugs, or nutritional supplements that may alleviate muscle damage (e.g., antioxidants, polyphenols, omega-3 polyunsaturated fatty acids)
3. Frequently engage in massage, cryotherapy, or other physiotherapeutic aids
4. Have a chronic disease or acute illness
5. Are pregnant or breast-feeding
6. Are smokers

### Ethical approval

This study protocol has received approval by the Department of Sport and Exercise Sciences Research Ethics Committee at Durham University (SPORT-2020-11-14T11_22_22-vlcz52; May 2021) and the Tyne and Wear South NHS Research Ethics Committee (21/NE/0073; May 2021). Informed written consent will be obtained from participants prior to any experimental procedures. This study has been registered at ClinicalTrials.gov (ID: NCT05111054).

### Randomisation

Following an initial virtual screening questionnaire and baseline assessments, participants will be randomised to an experimental condition (low-load or high-load resistance exercise). Folded pieces of paper labelled either ‘LL’ (low-load) or ‘HL’ (high-load) will be placed into an opaque envelope, and one will be drawn by the participant to determine their exercise condition. To ensure an equal number of males and females in each group, separate envelopes will be used, i.e., the ‘male’ envelope will contain 12 papers labelled ‘LL’ and 10 ‘HL’, as will the ‘female’ envelope. Due to the nature of the trial (i.e., varied intensity resistance exercise) it is not possible to blind the participant nor researcher to the experimental conditions.

## Protocol

### Baseline assessments and familiarisation

After the initial virtual screening, female participants will be asked to record their menstrual cycle and inform the lead researcher at the onset of menses. This information will be used to estimate the timing of the late follicular phase of the menstrual cycle, during which all baseline and experimental measures will be conducted.

During the first laboratory visit (-28 d), participants will arrive in the morning in a post-prandial state and complete a standard health and readiness to exercise questionnaire. Next, the correct form for use of the leg extension and leg curl resistance exercise machines (Versa leg extension/leg curl, Matrix, Wisconsin, USA) will be demonstrated to the participants before they commence the maximal strength test as previously described [70]. Briefly, participants will first complete a warm-up set with a light load, such that 10 repetitions can be easily performed. The exercise load will then progressively be increased by 10–20% for each successive attempt at performing a single repetition with the correct form. A rest period of 2 min will be allocated between attempts. Following a failed attempt, the exercise load will be reduced by 5–10% until the participant’s 1RM is established. This protocol will be completed for leg extension first and then for leg curl, with 5 min rest in between exercises. Utilising the 1RM protocol to assess maximal strength has been validated with a heterogenous population and is highly correlated with isometric and isokinetic peak torque using dynamometry for leg extension (r = 0.78 to 0.88) [71]. The test-retest reliability of the 1RM protocol is good to excellent (median ICC = 0.97; CV = 4.2%), independent of the sex, age, and training experience of the population [72].

Three days later (-25 d), participants will return to the laboratory in the morning following an overnight fast and abstinence from vigorous exercise and alcohol during the prior 24 h. Firstly, body mass and stature measurements will be obtained in light clothing. Female participants will confirm the absence of pregnancy, then whole-body composition will be assessed using dual energy X-ray absorptiometry (DXA) (GE Lunar iDXA, GE Healthcare, Madison, WI). Participants will be asked to consume 500 mL of water upon waking to ensure a euhydrated state, and to wear minimal and metal-free clothing (e.g., t-shirt and pair of shorts) during the scan, in accordance with the best practice procedures for DXA measurement [73, 74]. Total body mass, lean mass, fat mass, and body fat percentage will be measured. Following the assessment of body composition, the maximal strength test will be repeated, as per the protocol described above, to confirm the participants’ 1RM.

### Activity and dietary control

During the 3 wk rest period that will follow baseline measures, participants will be instructed to record their habitual activity and dietary intake for 3 d. This information will be used to examine any between-group differences. For each subsequent visit during the experimental period (+0, +24, +48, +72, and +168 h), participants will be prescribed a standardised breakfast (125 g low-fat fruit yoghurt, 2 slices of wholemeal bread/toast with jam/honey, and 200 mL semi-skimmed milk; 443 kcal, 20 g protein, 73 g carbohydrate, 7 g fat) to consume at home ~3 h prior to attending the laboratory. Otherwise, participants will consume their habitual diet, and a 24 h dietary recall will be conducted during each visit to identify any within- and between- group differences. Participants will be instructed to abstain from the use of non-steroidal anti-inflammatory drugs, strenuous exercise, engagement in massage or cryotherapy, and the consumption of alcohol, protein supplements, anti-oxidant vitamins, omega-3 polyunsaturated fatty acids, and ergogenic aids during the study period. All dietary intake data will be analysed using Nutritics software (NutriticsLTD, Swords, Ireland).

### Resistance exercise protocol

All resistance exercise sessions will be supervised. The described protocol will be performed on the leg extension machine followed by the leg curl machine, separated by 5 min rest. Participants will first perform a warm-up set consisting of 10 repetitions at 50% of their pre-determined 1RM. After 2 min rest, participants will complete 3 sets at their allocated exercise load (30% or 80% 1RM) each performed to volitional failure (defined as when a full repetition cannot be completed). A rest period of 2 min will be awarded between sets. A lifting tempo target of 1 and 2 s for concentric and eccentric phases of muscle contraction, respectively, will be set. Strong verbal encouragement will be given to all participants during exercise to ensure volitional failure is achieved.

### Muscle damage markers

The following assessments of muscle damage will be conducted in the same order during each visit (pre, post, 24, 48, 72, 168 h post-exercise) for all participants.

*Blood sampling*. Blood samples will be collected from an antecubital vein of the forearm using standard venepuncture techniques into three reagent-free vacutainers (3 × 10 mL). Samples will be left at room temperature for 30 min before being stored on ice and will later be analysed for serum concentrations of creatine kinase,interleukin-6, and oestrogen. Peak elevations in the serum concentration of creatine kinase–an intramuscular enzyme–are typically measured 2–5 d following EIMD and indicate an increase in sarcolemma permeability. Interleukin-6 is a pro-inflammatory cytokine and its elevated serum concentration within proximity to exercise (0–6 h) suggests acute inflammation and marks the secondary muscle damage response. Oestrogen may have a protective effect against EIMD, although the evidence in humans is not substantial.

#### Limb circumference

An increase in limb circumference following exercise is indicative of muscle swelling and may be used as a proxy measure of inflammation. Participants’ limb circumference will be measured using a standard anthropometric measuring tape at the mid, lower-, and upper- quartile points of the trochanterion-tibiale lateral site with the participant in a standing position. The mean value of the 3 sites will be used for the analysis.

#### Range of motion (ROM)

Participants’ ROM of the knee joint will be calculated as the difference between the relaxed and flexed knee joint angle, as measured using a standard goniometer with the participant in a supine position.

#### Muscle soreness

Participants will first rate their muscle soreness using a 10-point visual analogue scale (VAS) ranging from ‘not sore at all’ to ‘extremely sore’ while performing a simple bodyweight squat. Secondly, muscle soreness will be assessed with the pressure-pain threshold (PPT) test using a computerised pressure algometer (Medoc, AlgoMed, Ramat Yishai, Israel) with the participant in a supine position. The probe head (1 cm^2^) of the algometer will be placed at the mid, lower-, and upper- quartile points of the trochanterion-tibiale lateral site and increasing pressure will be applied until the participant indicated pain. The mean value of the 3 sites will be used for the analysis.

#### Maximal strength

The 1RM test as previously described will be repeated at 72 and 168 h only with leg extension and leg curl exercises.

### Serum preparation and analysis

Whole-blood samples will be centrifuged at 4°C, 4000 rpm for 15 min within 2 h of collection. Serum samples will be transferred into 1.5 mL microcentrifuge tubes and stored at -80°C until subsequent analysis. The concentrations of creatine kinase, interleukin-6, and oestrogen within serum will be determined with commercially available enzyme-linked immunosorbent assay (ELISA) kits.

### Statistical analysis

Statistical analysis will be conducted using IBM SPSS (version 25, SPSS Inc., Chicago, IL). All assumptions for statistical models will be assessed using the Shapiro-Wilk and Kolmogorov-Smirnov tests, and data that violate the assumptions will be log transformed prior to analysis. Independent *t*-tests with Bonferroni corrections will be used to examine any between-group differences in baseline characteristics, including body composition and habitual activity. The Levene’s test will be used to check for equality of variances between groups. A two-way mixed design analysis of variance will be used to analyse all muscle damage markers and dietary intake data between exercise conditions (low-load male, low-load female, high-load male, and high-load female) and within time points (-28 d, -25 d, pre, post, +24, +48, +72, and +168 h). Data sphericity will be assessed with the Mauchly’s test and any data that violates the Greenhouse-Geisser assumptions will be corrected with Huynh-Feldt. Any significant group × time interactions will be analysed *post hoc* using independent *t*-tests with Bonferroni corrections for between-group comparisons at each time point. Within-group differences across time will be analysed using paired *t*-tests. The within-group mean change from baseline to each time-point will be reported for each muscle damage marker to allow for future calculation of effect sizes and inclusion in meta-analyses. Stepwise multiple regression analyses will be conducted with age, sex, exercise intensity, hormonal contraceptive use, habitual activity, habitual daily protein intake, and body composition outcomes as the independent variables to identify predictors and confounders of each muscle damage marker. Statistical significance will be set at *P* < 0.05. Confidence intervals assume 95% confidence in the range of the mean. All data will be reported as mean ± standard deviation (SD) unless otherwise stated.

### Data storage and dissemination of findings

In order to ensure data protection, digital participant information will be stored on a password protected computer and data collection papers will be stored in a locked filing cabinet at the study site, available only to the primary investigators. Participant anonymity will be maintained through use of coded identification numbers, and all identifiable data will be destroyed following analysis of the complete dataset. Anonymised data will be uploaded to the institution’s repository to be available for secondary analysis. The study findings will be disseminated by publications in peer-reviewed journals and conferences.

## Discussion

This paper presents the study protocol for a randomised parallel-groups trial examining the impact of resistance exercise load on various indirect markers of muscle damage in untrained male and female young adults. Key aspects of the target population, exercise protocol, muscle damage markers, and time-points of assessment have been detailed. Previous reports on the impact of resistance exercise load on EIMD have produced equivocal findings, which may partly be due to inter-study differences in methodological design. Nonetheless, no study has yet considered the influence of sex. The proposed study aims to provide a direct sex comparison in the muscle damage response to low-load and high-load resistance exercise. It is hypothesised that the high-load resistance exercise will induce more marked changes in muscle damage markers in both males and females, though it cannot be ascertained at this time whether sex differences will be present. The outcomes of this study may inform the development of sex-specific training recommendations to maximise exercise adaptations while minimising muscle damage.

Advancing this work could further inform exercise training recommendations by expanding the study outcomes and considering alternative target populations and exercise protocols. For example, the present protocol includes limited analysis of serum biomarkers (creatine kinase and interleukin-6). Additional serum markers of muscle damage, such as myoglobin; skeletal troponins; and myosin heavy chain fragments, and of inflammation, such as C-reactive protein; tumor necrosis factor-α; and various interleukins could be analysed to corroborate the biochemical response to exercise. It may also be advantageous to obtain more frequent serum samples during the early post-exercise recovery period (i.e., 0–24 h) when some inflammatory markers reach peak concentration. Here we propose that muscle swelling–an indicator of inflammation–be conveniently assessed via limb circumference measurements. However, if available, more advanced techniques for assessing muscle swelling e.g., ultrasound imaging would be preferential and would provide additional information on muscle cross-sectional area, thickness, volume, pennation angle, and fascicle length.

We propose that leg-based resistance exercise be performed to induce muscle damage, though alternatively, the impact of upper- or whole- body resistance exercise with varied loads could be explored. Following EIMD, the present protocol excludes the measurement of maximal voluntary contraction during the early post-exercise recovery period. This is suggested to avoid further muscle damage being induced by the 1RM test and to better reflect real-life exercise practice, in which a day or two of rest would ensue the initial exercise bout. However, it is appreciated that maximal strength is most impaired immediately following a damaging exercise bout and thus, this time-point may wish to be captured. While we propose that maximal strength be determined by a 1RM test (due to equipment availability), the use of isokinetic dynamometry could alternatively be applied. Further, the addition of other exercise performance measures, for example countermovement jump height/power, agility, and sport-specific actions could benefit this work.

Finally, the application of this research to wider populations could be achieved through its replication in diverse study samples. Conducting this research in untrained male and female young adults is a pragmatic starting point, though comparing between male and female older adults or between trained and untrained females is suggested as future direction. The present research protocol may be used to guide the design of future studies aiming to bridge the sex data gap and inform sex-specific exercise recommendations.

## Supporting information

S1 File (PDF)

S2 File (PDF)

S1 Checklist SPIRIT 2013 checklist: Recommended items to address in a clinical trial protocol and related documents*. (DOC)
