# Supplementary material for: Sex differences in the impact of resistance exercise load on muscle damage: A protocol for a randomised parallel group trial
Source: PLoS One. 2022 Sep 29;17(9):e0275221. doi: 10.1371/journal.pone.0275221 (PMC9521925; doi:10.1371/journal.pone.0275221)
Supplement: S1 File — (PDF) [file pone.0275221.s001.pdf]

ClinicalTrials.gov PRS
Protocol Registration and Results System

ClinicalTrials.gov Protocol Registration and Results System (PRS) Receipt
Release Date: November 8, 2021

ClinicalTrials.gov ID: NCT05111054

Study Identification

Unique Protocol ID: 290580-LOAD

Brief Title: Sex Differences in Muscle Damage Following Resistance Exercise at Low or High Intensity (EIMD-LOAD)

Official Title: Sex Differences in Resistance Exercise-induced Muscle Damage: The Impact of Exercise Load

Secondary IDs:

Study Status

Record Verification: November 2021

Overall Status: Suspended [Time availability within PhD studentship]

Study Start: January 2023 [Anticipated]

Primary Completion: January 2024 [Anticipated]

Study Completion: January 2024 [Anticipated]

Sponsor/Collaborators

Sponsor: Durham University

Responsible Party: Principal Investigator
Investigator: Alice Pearson [apearson]
Official Title: Principal Investigator
Affiliation: Durham University

Collaborators:

Oversight

U.S. FDA-regulated Drug: No

U.S. FDA-regulated Device: No

U.S. FDA IND/IDE: No

Human Subjects Review: Board Status: Approved
Approval Number: 21/NE/0073
Board Name: Tyne & Wear South Research Ethics Committee
Board Affiliation: Health Research Authority, NHS
Phone:
Email: tyneandwearsouth.rec@hra.nhs.uk
Address:

NHSBT Newcastle Blood Donor Centre
Holland Drive
Newcastle Upon Tyne
NE2 4NQ

Data Monitoring:

Study Description

Brief Summary: Purpose: To investigate the impact of exercise load on resistance exercise-induced muscle damage in untrained males and females.

Rationale: Unaccustomed resistance exercise can cause muscle damage, presenting as muscle soreness and reduced muscle function - such as loss of strength, power, and flexibility - for several days after the exercise bout. Therefore, individuals may require longer recovery periods before performing another exercise bout, and their performance may be impaired. Further, muscle soreness may reduce exercise compliance, particularly in novice individuals. Over time, this may compromise the gains in muscle mass and strength achieved through exercise training. Therefore, strategies to reduce the severity of exercise-induced muscle damage and/or to enhance post-exercise recovery processes are advantageous for exercising individuals.

One such strategy is to perform resistance exercise with lighter loads, i.e. <70% one repetition maximum (1RM). Low-load resistance training has shown to induce comparable gains in muscle mass and strength to high-load (≥70% 1RM), while being perceptively less exerting. Low-load resistance exercise may place less mechanical stress on muscle fibres and accordingly, its impact on muscle damage has been investigated. While several studies have reported less severe muscle damage, muscle soreness, and functional impairments with low-load resistance exercise compared to high-load, others have found no differences. Further, there is a lack of studies conducted solely in females or comparing between sexes. It has been suggested that males and females respond differently to muscle damage, and therefore, this research aims to provide a sex comparison in the muscle damage response to an acute bout of resistance exercise performed with low or high loads.

Therefore, 40 healthy, young (18-35 years) adults (20 males, 20 females) will be recruited to participate in this randomised controlled trial. Maximal leg strength and body composition (by dual-energy X-ray absorptiometry; DXA) will be conducted at baseline. In females, all primary outcome measures will be obtained during the late follicular phase of the menstrual cycle. Participants will then be randomised to a low-load (30% 1RM) or high-load (80% 1RM) exercise condition.

Three weeks later, participants will complete a resistance exercise session at their allocated intensity on leg extension and leg curl machines to induce muscle damage. Various measures of muscle damage (blood biomarkers, muscle soreness, flexibility, and swelling) will be obtained before, immediately after, and 24, 48, 72, and 168 h after the exercise protocol. The maximal strength test will be repeated 72 and 168 h after the exercise. Participants' habitual activity and dietary intake will be monitored and controlled throughout the study period.

Expected outcome: It is expected that the resistance exercise protocol will induce muscle damage, which will be less severe in the low-load exercise condition. It cannot be ascertained whether males and females will have the same responses to the exercise.

Detailed Description:

Conditions

Conditions: Muscle Damage

Keywords:

Study Design

Study Type: Interventional

Primary Purpose: Basic Science

Study Phase: N/A

Interventional Study Model: Parallel Assignment

Number of Arms: 2

Masking: None (Open Label)

Allocation: Randomized

Enrollment: 40 [Anticipated]

Arms and Interventions

Arms	Assigned Interventions
Experimental: Low-Load<br>Acute resistance exercise performed at 30% 1RM	Resistance Exercise<br>Acute leg-based resistance exercise bout (3 sets performed to volitional failure on leg extension and leg curl machines)
Active Comparator: High-Load<br>Acute resistance exercise performed at 80% 1RM	Resistance Exercise<br>Acute leg-based resistance exercise bout (3 sets performed to volitional failure on leg extension and leg curl machines)

Outcome Measures

Primary Outcome Measure:

1. Maximal Voluntary Contraction at baseline
   One-repetition maximum (1RM) test: leg extension and leg curl machines

   [Time Frame: Baseline]
2. Change from baseline Maximal Voluntary Contraction at 72-hours post-exercise
   One-repetition maximum (1RM) test: leg extension and leg curl machines

   [Time Frame: 72-hours after the exercise bout]
3. Change from baseline Maximal Voluntary Contraction at 168-hours post-exercise
   One-repetition maximum (1RM) test: leg extension and leg curl machines

   [Time Frame: 168-hours after the exercise bout]
4. Creatine kinase concentration at baseline
   Serum concentration of creatine kinase from venous blood sampling

   [Time Frame: Immediately pre-exercise]
5. Change from baseline in Creatine Kinase concentration immediately post-exercise
   Serum concentration of creatine kinase from venous blood sampling

   [Time Frame: Immediately after the exercise bout]

6. Change from baseline in Creatine Kinase concentration at 24-hours post-exercise
   Serum concentration of creatine kinase from venous blood sampling

   [Time Frame: 24-hours after the exercise bout]
7. Change from baseline in Creatine Kinase concentration at 48-hours post-exercise
   Serum concentration of creatine kinase from venous blood sampling

   [Time Frame: 48-hours after the exercise bout]
8. Change from baseline in Creatine Kinase concentration at 72-hours post-exercise
   Serum concentration of creatine kinase from venous blood sampling

   [Time Frame: 72-hours after the exercise bout]
9. Change from baseline in Creatine Kinase concentration at 168-hours post-exercise
   Serum concentration of creatine kinase from venous blood sampling

   [Time Frame: 168-hours after the exercise bout]
10. Interleukin-6 concentration at baseline
    Serum concentration of Interleukin-6 from venous blood sampling

    [Time Frame: Immediately pre-exercise]
11. Change from baseline in Interleukin-6 concentration immediately post-exercise
    Serum concentration of Interleukin-6 from venous blood sampling

    [Time Frame: Immediately after the exercise bout]
12. Change from baseline in Interleukin-6 concentration at 24-hours post-exercise
    Serum concentration of Interleukin-6 from venous blood sampling

    [Time Frame: 24-hours after the exercise bout]
13. Change from baseline in Interleukin-6 concentration at 48-hours post-exercise
    Serum concentration of Interleukin-6 from venous blood sampling

    [Time Frame: 48-hours after the exercise bout]
14. Change from baseline in Interleukin-6 concentration at 72-hours post-exercise
    Serum concentration of Interleukin-6 from venous blood sampling

    [Time Frame: 72-hours after the exercise bout]
15. Change from baseline in Interleukin-6 concentration at 168-hours post-exercise
    Serum concentration of Interleukin-6 from venous blood sampling

    [Time Frame: 168-hours after the exercise bout]
16. Muscle soreness (pressure algometry) at baseline
    Self-perceived rating of muscle soreness with use of pressure algometry

    [Time Frame: Immediately pre-exercise]
17. Change in muscle soreness (pressure algometry) immediately post-exercise
    Self-perceived rating of muscle soreness with use of pressure algometry

    [Time Frame: Immediately after the exercise bout]
18. Change in muscle soreness (pressure algometry) at 24-hours post-exercise
    Self-perceived rating of muscle soreness with use of pressure algometry

    [Time Frame: 24-hours after the exercise bout]
19. Change in muscle soreness (pressure algometry) at 48-hours post-exercise
    Self-perceived rating of muscle soreness with use of pressure algometry

    [Time Frame: 48-hours after the exercise bout]
20. Change in muscle soreness (pressure algometry) at 72-hours post-exercise
    Self-perceived rating of muscle soreness with use of pressure algometry

[Time Frame: 72-hours after the exercise bout]

21. Change in muscle soreness (pressure algometry) at 168-hours post-exercise
Self-perceived rating of muscle soreness with use of pressure algometry

[Time Frame: 168-hours after the exercise bout]

22. Muscle soreness (visual analogue scale, VAS) at baseline
Self-perceived rating of muscle soreness while performing a bodyweight squat with use of a visual analogue scale (0 - not sore at all, 10 - extremely sore)

[Time Frame: Immediately pre-exercise]

23. Change in muscle soreness (visual analogue scale, VAS) immediately post-exercise
Self-perceived rating of muscle soreness while performing a bodyweight squat with use of a visual analogue scale (0 - not sore at all, 10 - extremely sore)

[Time Frame: Immediately after the exercise bout]

24. Change in muscle soreness (visual analogue scale, VAS) at 24-hours post-exercise
Self-perceived rating of muscle soreness while performing a bodyweight squat with use of a visual analogue scale (0 - not sore at all, 10 - extremely sore)

[Time Frame: 24-hours after the exercise bout]

25. Change in muscle soreness (visual analogue scale, VAS) at 48-hours post-exercise
Self-perceived rating of muscle soreness while performing a bodyweight squat with use of a visual analogue scale (0 - not sore at all, 10 - extremely sore)

[Time Frame: 48-hours after the exercise bout]

26. Change in muscle soreness (visual analogue scale, VAS) at 72-hours post-exercise
Self-perceived rating of muscle soreness while performing a bodyweight squat with use of a visual analogue scale (0 - not sore at all, 10 - extremely sore)

[Time Frame: 72-hours after the exercise bout]

27. Change in muscle soreness (visual analogue scale, VAS) at 168-hours post-exercise
Self-perceived rating of muscle soreness while performing a bodyweight squat with use of a visual analogue scale (0 - not sore at all, 10 - extremely sore)

[Time Frame: 168-hours after the exercise bout]

28. Range of motion at baseline
Flexibility of the exercised limb as determined by goniometry

[Time Frame: Immediately pre-exercise]

29. Change in range of motion immediately post-exercise
Flexibility of the exercised limb as determined by goniometry

[Time Frame: Immediately after the exercise bout]

30. Change in range of motion at 24-hours post-exercise
Flexibility of the exercised limb as determined by goniometry

[Time Frame: 24-hours after the exercise bout]

31. Change in range of motion at 48-hours post-exercise
Flexibility of the exercised limb as determined by goniometry

[Time Frame: 48-hours after the exercise bout]

32. Change in range of motion at 72-hours post-exercise
Flexibility of the exercised limb as determined by goniometry

[Time Frame: 72-hours after the exercise bout]

33. Change in range of motion at 168-hours post-exercise
Flexibility of the exercised limb as determined by goniometry

[Time Frame: 168-hours after the exercise bout]

34. Limb circumference at baseline
Measure of leg circumference with use of standard anthropometric tape to indicate muscle swelling

[Time Frame: Immediately pre-exercise]

35. Change in limb circumference immediately post-exercise
Measure of leg circumference with use of standard anthropometric tape to indicate muscle swelling

[Time Frame: Immediately after the exercise bout]

36. Change in limb circumference at 24-hours post-exercise
Measure of leg circumference with use of standard anthropometric tape to indicate muscle swelling

[Time Frame: 24-hours after the exercise bout]

37. Change in limb circumference at 48-hours post-exercise
Measure of leg circumference with use of standard anthropometric tape to indicate muscle swelling

[Time Frame: 48-hours after the exercise bout]

38. Change in limb circumference at 72-hours post-exercise
Measure of leg circumference with use of standard anthropometric tape to indicate muscle swelling

[Time Frame: 72-hours after the exercise bout]

39. Change in limb circumference at 168-hours post-exercise
Measure of leg circumference with use of standard anthropometric tape to indicate muscle swelling

[Time Frame: 168-hours after the exercise bout]

Eligibility

Minimum Age: 18 Years

Maximum Age: 35 Years

Sex: All

Gender Based: No

Accepts Healthy Volunteers: Yes

Criteria: Inclusion Criteria:

- BMI 18.5 - 25.0 kg/m2
- Untrained in resistance exercise
- No known chronic disease or current acute illness
- No current or recent (past 3 months) musculoskeletal injury
- No frequent use (2x per week for past month) of non-steroidal anti-inflammatory drugs, anti-oxidant supplements, polyunsaturated omega-3 fatty acids (and other substances that may alleviate muscle damage) and compliant to abstain from use during experimental period
- No recent or current engagement in massage or cryotherapy and compliant to abstain from use during experimental period
- Females will be eumenorrheic (regular menstrual cycle) >12 months
- Absence of pregnancy and breast-feeding

Exclusion Criteria:

- Underweight
- Overweight/obese
- Resistance trained
- Current or recent injury
- Pregnancy or breast-feeding
- Unwilling to provide blood samples, perform resistance exercise, or abstain from use of NSAID's and other substances (stated above)

- Unwilling to abstain from other forms of exercise during the experimental period

Contacts/Locations

Central Contact Person: Alice G Pearson
Telephone: 07771357222
Email: alice.g.pearson@durham.ac.uk

Central Contact Backup: Lindsay S Macnaughton
Email: lindsay.s.macnaughton@durham.ac.uk

Study Officials: Alice G Pearson
Study Principal Investigator
Durham University

Locations: **United Kingdom**
Durham University, The Graham Sports Centre
Durham, County Durham, United Kingdom, DH1 3HN
Contact: Alice G Pearson 07771357222 alice.g.pearson@durham.ac.uk
Contact: Lindsay S Macnaughton lindsay.s.macnaughton@durham.ac.uk

IPDSharing

Plan to Share IPD:

References

Citations:

Links:

Available IPD/Information:

U.S. National Library of Medicine | U.S. National Institutes of Health | U.S. Department of Health & Human Services
