# Supplementary material for: Sex differences in the impact of resistance exercise load on muscle damage: A protocol for a randomised parallel group trial
Source: PLoS One. 2022 Sep 29;17(9):e0275221. doi: 10.1371/journal.pone.0275221 (PMC9521925; doi:10.1371/journal.pone.0275221)
Supplement: S2 File — (PDF) [file pone.0275221.s002.pdf]

Welcome to the Integrated Research Application System

IRAS Project Filter

The integrated dataset required for your project will be created from the answers you give to the following questions. The system will generate only those questions and sections which (a) apply to your study type and (b) are required by the bodies reviewing your study. Please ensure you answer all the questions before proceeding with your applications.

Please complete the questions in order. If you change the response to a question, please select 'Save' and review all the questions as your change may have affected subsequent questions.

Please enter a short title for this project (maximum 70 characters)
Sex Differences in Muscle Damage: Exercise Load & Protein Feeding (v1)

1. Is your project research?

(•) Yes ( ) No

2. Select one category from the list below:

- ( ) Clinical trial of an investigational medicinal product
- ( ) Clinical investigation or other study of a medical device
- ( ) Combined trial of an investigational medicinal product and an investigational medical device
- ( ) Other clinical trial to study a novel intervention or randomised clinical trial to compare interventions in clinical practice
- (•) Basic science study involving procedures with human participants
- ( ) Study administering questionnaires/interviews for quantitative analysis, or using mixed quantitative/qualitative methodology
- ( ) Study involving qualitative methods only
- ( ) Study limited to working with human tissue samples (or other human biological samples) and data (specific project only)
- ( ) Study limited to working with data (specific project only)
- ( ) Research tissue bank
- ( ) Research database

If your work does not fit any of these categories, select the option below:

( ) Other study

2a. Will the study involve the use of any medical device without a UKCA/CE UKNI/CE Mark, or a UKCA/CE UKNI/CE marked device which has been modified or will be used outside its intended purposes?

( ) Yes (•) No

2b. Please answer the following question(s):

a) Does the study involve the use of any ionising radiation? (•) Yes ( ) No

- Does the study involve exposure to radioactive materials? ( ) Yes (•) No

b) Will you be taking new human tissue samples (or other human biological samples)? (•) Yes ( ) No

c) Will you be using existing human tissue samples (or other human biological samples)? ( ) Yes (•) No

d) Will the study involve any other clinical procedures with participants (e.g. MRI, ultrasound, physical examination)? (•) Yes ( ) No

3. In which countries of the UK will the research sites be located?*(Tick all that apply)*

- [x] England
- [ ] Scotland
- [ ] Wales
- [ ] Northern Ireland

3a. In which country of the UK will the lead NHS R&D office be located:

( ) England

( ) Scotland

( ) Wales

( ) Northern Ireland

(•) This study does not involve the NHS

4. Which applications do you require?

- [ ] NHS/HSC Research and Development offices
- [ ] Social Care Research Ethics Committee
- [x] Research Ethics Committee
- [ ] Confidentiality Advisory Group (CAG)
- [ ] Her Majesty's Prison and Probation Service (HMPPS)

5. Will any research sites in this study be NHS organisations?

( ) Yes (•) No

6. Do you plan to include any participants who are children?

( ) Yes (•) No

7. Do you plan at any stage of the project to undertake intrusive research involving adults lacking capacity to consent for themselves?

( ) Yes (•) No

Answer Yes if you plan to recruit living participants aged 16 or over who lack capacity, or to retain them in the study following loss of capacity. Intrusive research means any research with the living requiring consent in law. This includes use of identifiable tissue samples or personal information, except where application is being made to the Confidentiality Advisory Group to set aside the common law duty of confidentiality in England and Wales. Please consult the guidance notes for further information on the legal frameworks for research involving adults lacking capacity in the UK.

8. Do you plan to include any participants who are prisoners or young offenders in the custody of HM Prison Service or who are offenders supervised by the probation service in England or Wales?

( ) Yes (•) No

9. Is the study or any part of it being undertaken as an educational project?

(•) Yes ( ) No

Please describe briefly the involvement of the student(s):
The primary researcher is a second year PhD student at Durham University. She will be responsible for participant recruitment, all data collection and storage, under the supervision of Dr Karen Hind and Dr Lindsay MacNaughton.

9a. Is the project being undertaken in part fulfilment of a PhD or other doctorate?

(•) Yes ( ) No

10. Will this research be financially supported by the United States Department of Health and Human Services or any of its divisions, agencies or programs?

( ) Yes (•) No

11. Will identifiable patient data be accessed outside the care team without prior consent at any stage of the project (including identification of potential participants)?

( ) Yes (•) No

Integrated Research Application System
Application Form for Basic science study involving procedures with human participants

NHS
Health Research Authority

Application to NHS/HSC Research Ethics Committee

The Chief Investigator should complete this form. Guidance on the questions is available wherever you see this symbol displayed. We recommend reading the guidance first. The complete guidance and a glossary are available by selecting Help.

Please define any terms or acronyms that might not be familar to lay reviewers of the application.

Short title and version number: (maximum 70 characters - this will be inserted as header on all forms)
Sex Differences in Muscle Damage: Exercise Load & Protein Feeding (v1)

Please complete these details after you have booked the REC application for review.

REC Name:

REC Reference Number: **Submission date:**

PART A: Core study information

1. ADMINISTRATIVE DETAILS

A1. Full title of the research:

Sex differences in muscle damage following acute resistance exercise: The impact of load intensity and dietary protein feeding

A2-1. Educational projects

Name and contact details of student(s):

Student 1

	Title	Forename/Initials	Surname
	Miss	Alice	Pearson

Address 21 Pine Avenue

Durham

Post Code DH1 2EX
E-mail alice.g.pearson@durham.ac.uk
Telephone 07771357222
Fax

Give details of the educational course or degree for which this research is being undertaken:

Name and level of course/ degree:
PhD Human Nutrition & Exercise Physiology

Name of educational establishment:
Durham University

Name and contact details of academic supervisor(s):

Academic supervisor 1

	Title	Forename/Initials	Surname
	Dr	Karen	Hind
Address	42 Old Elvet		
	Durham		
Post Code	DH1 3HN		
E-mail	karen.hind@durham.ac.uk		
Telephone			
Fax			

Academic supervisor 2

	Title	Forename/Initials	Surname
	Dr	Lindsay	Macnaughton
Address	42 Old Elvet		
	Durham		
Post Code	DH1 3HN		
E-mail	lindsay.s.macnaughton@durham.ac.uk		
Telephone			
Fax			

Please state which academic supervisor(s) has responsibility for which student(s):
Please click "Save now" before completing this table. This will ensure that all of the student and academic supervisor details are shown correctly.

**Student(s)**	**Academic supervisor(s)**
**Student 1** Miss Alice Pearson	☑ Dr Karen Hind<br>☑ Dr Lindsay Macnaughton

A copy of a current CV for the student and the academic supervisor (maximum 2 pages of A4) must be submitted with the application.

A2-2. Who will act as Chief Investigator for this study?

- (●) Student
- ( ) Academic supervisor
- ( ) Other

A3-1. Chief Investigator:

	Title	Forename/Initials	Surname
	Miss	Alice	Pearson
Post	PhD Researcher		
Qualifications	MSc., SENr.		
ORCID ID	0000 0002 8640 1512		
Employer	Durham University		
Work Address	42 Old Elvet		
	Durham		
Post Code	DH1 3HN		
Work E-mail	alice.g.pearson@durham.ac.uk		
* Personal E-mail	alice.g.pearson@gmail.com		
Work Telephone			
* Personal Telephone/Mobile	07771357222		
Fax			

** This information is optional. It will not be placed in the public domain or disclosed to any other third party without prior consent.*
A copy of a current CV (maximum 2 pages of A4) for the Chief Investigator must be submitted with the application.

A4. Who is the contact on behalf of the sponsor for all correspondence relating to applications for this project?
This contact will receive copies of all correspondence from REC and HRA/R&D reviewers that is sent to the CI.

	Title	Forename/Initials	Surname
	Miss	Alice	Pearson
Address	21 Pine Avenue		
	Durham		
Post Code	DH1 2EX		
E-mail	alice.g.pearson@durham.ac.uk		
Telephone	07771357222		
Fax			

A5-1. Research reference numbers. *Please give any relevant references for your study:*

Applicant's/organisation's own reference number, e.g. R & D (if available):

Sponsor's/protocol number:

Protocol Version:

Protocol Date:

Funder's reference number (enter the reference number or state not applicable):

Project website:

Registry reference number(s):
The UK Policy Framework for Health and Social Care Research sets out the principle of making information about research publicly available. Furthermore: Article 19 of the World Medical Association Declaration of Helsinki adopted in 2008 states that "every clinical trial must be registered on a publicly accessible database before recruitment of the

first subject"; and the International Committee of Medical Journal Editors (ICMJE) will consider a clinical trial for publication only if it has been registered in an appropriate registry. Please see guidance for more information.

International Standard Randomised Controlled Trial Number (ISRCTN):

ClinicalTrials.gov Identifier (NCT number):

Additional reference number(s):

Ref.Number Description	Reference Number

A5-2. Is this application linked to a previous study or another current application?

○ Yes ◉ No

Please give brief details and reference numbers.

2. OVERVIEW OF THE RESEARCH

To provide all the information required by review bodies and research information systems, we ask a number of specific questions. This section invites you to give an overview using language comprehensible to lay reviewers and members of the public. Please read the guidance notes for advice on this section.

A6-1. Summary of the study. *Please provide a brief summary of the research (maximum 300 words) using language easily understood by lay reviewers and members of the public. Where the research is reviewed by a REC within the UK Health Departments' Research Ethics Service, this summary will be published on the Health Research Authority (HRA) website following the ethical review. Please refer to the question specific guidance for this question.*

Unaccustomed resistance exercise can cause damage to muscle fibres that may consequently lead to muscle soreness, loss of strength, and reduced flexibility for several days following the exercise bout. These impairments prolong recovery time and likely impact exercise performance, which over time, may limit the gains in muscle mass and strength associated with resistance training. Therefore, establishing strategies to limit exercise-induced muscle damage will be advantageous to exercising individuals. This research will investigate the impact of exercise intensity in both sexes (part A) and dietary protein feeding (part B) as potential strategies for reducing muscle damage.

For part A of the research, young, healthy males and females (n = 40) will be recruited. Participants will perform a single bout of leg-based resistance exercise using either heavy or lighter weights. Muscle damage will be assessed before, immediately after, and in 24-hour intervals after the exercise through indirect blood markers (from blood samples), self-perceived muscle soreness (via pressure algometry), and muscle function (strength and flexibility). Muscle damage will be compared between sexes and exercise loads, and related to total lean body mass, as determined by dual energy X-ray absorptiometry (DXA) at baseline.

For part B of the research, young, healthy females (n = 22) will be recruited. Participants will perform a single bout of high-intensity leg-based resistance exercise. Before the exercise, and every 4 hours afterwards for 3 days participants will consume either 20 g of milk protein or a carbohydrate-based placebo. Previous research has shown that milk protein effectively reduces muscle soreness, helps maintain strength, and lowers the concentration of damage and inflammatory biomarkers in males, but this has not yet been investigated in untrained females. Muscle damage will be assessed as per part A and compared between the protein and placebo groups and related to baseline body composition, as determined by DXA.

A6-2. Summary of main issues. *Please summarise the main ethical, legal, or management issues arising from your study and say how you have addressed them.*

Not all studies raise significant issues. Some studies may have straightforward ethical or other issues that can be identified and managed routinely. Others may present significant issues requiring further consideration by a REC, R&D office or other review body (as appropriate to the issue). Studies that present a minimal risk to participants may raise complex organisational or legal issues. You should try to consider all the types of issues that the different reviewers may need to consider.

Collection of blood samples may cause discomfort, minor bruising, and carries a risk of infection - blood samples will be collected by a trained phlebotomist wearing the correct personal protective equipment (e.g. disposable gloves,

laboratory coat). All surfaces (e.g. chair, tables) will be cleaned before and after use and the researcher will practice hand washing prior to touching any equipment.

Performing the resistance exercise may cause musculoskeletal injury - A readiness to exercise questionnaire will be completed prior to testing and participants will not be eligible to participate if they have a current or previous (past 3 months) musculoskeletal injury. Participants will be demonstrated correct lifting technique; perform an appropriate warm-up; and be supervised at all times.

Performing the Pressure-Pain Threshold test with a pressure algometer may cause discomfort and minor bruising - The participant will be instructed to indicate when the pressure applied with the algometer becomes painful, at which point, the pressure will be released. The test will be performed on alternate legs to reduce bruising of the same site and participants will be advised to treat any bruising with rest, ice, compression, and elevation.

Assessing body composition by dual energy X-ray absorptiometry exposes the participant to ionising radiation. The effective dose received per scan is minimal and considered safe at 0.002 mSv, which is the equivalent of a few days of natural background radiation in the UK. Multiple DXA scans are considered safe to both the participant and researcher.

There is a risk of spreading or being infected with Covid-19. The laboratory has a maximum occupancy that will be adhered to; a 2 metre distance will be maintained where possible; where close contact is required e.g. when taking blood samples, performing the pressure-pain threshold test, and measuring leg circumference, a face covering will be worn by both the researcher and participant and the researcher will wear disposable gloves; all surfaces including the exercise equipment will be cleaned before and after use with the correct cleaning solution; frequent hand-washing will be performed; and if either a participant or the researcher develops Covid-19 symptoms they will be instructed not to attend the laboratory and to self-isolate in line with government guidance.

3. PURPOSE AND DESIGN OF THE RESEARCH

A7. Select the appropriate methodology description for this research. *Please tick all that apply*:

- [ ] Case series/ case note review
- [ ] Case control
- [ ] Cohort observation
- [ ] Controlled trial without randomisation
- [ ] Cross-sectional study
- [ ] Database analysis
- [ ] Epidemiology
- [ ] Feasibility/ pilot study
- [x] Laboratory study
- [ ] Metanalysis
- [ ] Qualitative research
- [ ] Questionnaire, interview or observation study
- [x] Randomised controlled trial
- [ ] Other (please specify)

A10. What is the principal research question/objective? *Please put this in language comprehensible to a lay person.*

Objective 1 (part A): To investigate sex differences in the muscle damage response to acute resistance exercise performed with heavy or lighter weights.

Objective 2 (part B): To investigate the impact of milk protein ingestion on resistance exercise-induced muscle damage and recovery in females.

A11. What are the secondary research questions/objectives if applicable? *Please put this in language comprehensible to*

a lay person.

To investigate whether a relationship exists between exercise-induced muscle damage and total lean body mass (muscle mass)

To conduct a precision assessment of dual energy X-ray absorptiometry for measuring whole body composition (part A)

A12. What is the scientific justification for the research? *Please put this in language comprehensible to a lay person.*

Unaccustomed resistance exercise can cause damage to muscle fibres. Subsequently, individuals may experience muscle soreness and reduced muscle function - such as loss of strength, power, and flexibility – for several days after the exercise bout. This means that individuals may need longer recovery periods before performing another exercise bout, and their performance may be impaired. Further, muscle soreness may reduce the desire to engage in subsequent exercise bouts, particularly in individuals unaccustomed to resistance exercise. Over time, this may compromise the gains in muscle mass and strength which can be achieved through repeated bouts of resistance exercise, i.e. training. Therefore, strategies aiming to reduce the severity of exercise-induced muscle damage and/or to enhance post-exercise recovery processes are advantageous for exercising individuals.

One strategy that may be beneficial in reducing the muscle damage response to resistance exercise is to lift lighter weights, i.e. <70% one repetition maximum (1RM; the maximum weight that can be lifted once). In the past decade, attention has been paid to low-load resistance training owing to its ability to increase muscle mass to the same extent as when heavier weights are lifted (≥70% 1RM) E.g. (Mitchell et al., 2012; Morton et al., 2016; Schoenfeld et al., 2016; Stefanaki et al., 2019). Additionally, individuals engaging in low-load resistance exercise have reported lower ratings of perceived exertion relative to heavier loads (Chen et al., 2020; Mendham et al., 2011). As such, exercising with lighter loads may be the preferred option for those unaccustomed to resistance exercise; elderly populations; and individuals recovering from musculoskeletal injury. Further, low-load resistance exercise may place less mechanical stress on muscle fibres and accordingly, its impact on muscle damage has been investigated. While several studies have reported less severe muscle damage, self-perceived muscle soreness, and functional impairments (such as loss of strength, power, and flexibility) with low-load resistance exercise compared to high-load (Chen et al., 2020; Hasenoehrl et al., 2017; Mendham et al., 2011; NOSAKA & NEWTON, 2002; Orssatto et al., 2018; P. M. Tiidus & Ianuzzo, 1983), others have found no differences (Draganidis et al., 2013; Güzel et al., 2007; Paschalis et al., 2005; Peake et al., 2006; Uchida et al., 2009). Further, the majority of studies to date have been conducted in young males, with a complete lack of data solely in females.

There is evidence to suggest that males and females may respond differently to resistance exercise, with respect to muscle damage. For instance, some studies have shown greater and more sustained increases in blood markers of muscle damage and inflammation in males than females following resistance exercise (Amorim et al., 2014; Heavens et al., 2014). It has been speculated whether these differences are due to a protective effect of oestrogens (Enns & Tiidus, 2008; Peter M Tiidus, 2003). Therefore, not only is the muscle damage response to heavy vs. lighter resistance exercise inconsistent in males, but it cannot be determined whether females will respond in the same way. Thus, the first objective (part a) of this research is to compare the muscle damage response induced by an acute bout of high- or low- load resistance exercise between males and females.

Another strategy that has shown to reduce exercise-induced muscle damage is the consumption of dietary protein, either as a whole-food source (such as milk) or as a supplement (such as isolated whey protein). It is believed that protein, or rather the amino acids which make up protein, aid the repair or replacement of damaged muscle fibres, then promote growth of the new healthy fibres. Accordingly, research has shown that ingesting protein/amino acids before or after muscle-damaging exercise can alleviate muscle soreness, improve blood markers of muscle damage, and reduce the decline in maximal force and flexibility. In particular, studies have shown that consuming a ~20 g dose of milk protein immediately after (Cockburn et al., 2008) or for several times a day after (Draganidis et al., 2017) resistance exercise improved the recovery time of muscle soreness and maximum force, and also lowered levels of damage markers in the blood. However, these studies were conducted with male participants who were well-trained in resistance exercise. It is not yet known whether milk protein effectively reduces exercise-induced muscle damage in untrained females. Therefore, the second objective (part b) of this research is to investigate the impact of milk protein ingestion on exercise-induced muscle damage in untrained females.

A13. Please summarise your design and methodology. *It should be clear exactly what will happen to the research participant, how many times and in what order. Please complete this section in language comprehensible to the lay person. Do not simply reproduce or refer to the protocol. Further guidance is available in the guidance notes.*

OBJECTIVE 1/PART A: This study will employ a randomised parallel groups design, for which 40 healthy participants (20 males and 20 females) aged 18-35 years will be recruited. Prior to the first laboratory visit, prospective participants will have the opportunity to ask questions and gain further information about the study via a video/phone call with the

lead researcher. If willing to participate, participants will be asked to electronically sign a consent form. Female participants will be asked to record their menstrual cycle and inform the lead researcher at the onset of menses – this information will be used to arrange the time of the baseline assessments, as all measures will be conducted during the late follicular phase of the menstrual cycle.

For the first visit, which acts as a familiarisation session, participants will arrive at the laboratory in the morning and be screened for eligibility with a standard health and readiness to exercise questionnaire. Next, participants will be familiarised with the exercise equipment and demonstrated correct form. Then, participants' maximal strength will be estimated though a one repetition maximum (1RM) test on the leg press and leg extension machines. Approximately three days later, participants will return to the laboratory and a baseline assessment of body composition will be conducted using dual energy X-ray absorptiometry (DXA). Two whole-body DXA scans will be conducted in each participant (one immediately following the other after repositioning) to allow for the precision of DXA to be measured. The 1RM test will be repeated on the leg press and leg extension machines for confirmation of maximal strength. Participants will then be randomly assigned to either a low-load (30% 1RM) or high-load (80% 1RM) exercise condition, such that each group will have an equal number of males and females.

Visit three will occur three weeks later. In between visits two and three, participants will be asked to record their habitual diet and activity for three days, which will be used to assess any between-group differences. For each subsequent assessment day, participants will be prescribed a standardised breakfast to consume at home 3 hours prior to attending the laboratory. Otherwise, participants will be instructed to consume their habitual diet and, during each laboratory visit, a 24-hour dietary recall will be conducted to assess any changes in dietary intake. Participants will be instructed to abstain from the use of non-steroidal anti-inflammatory drugs, strenuous exercise, engagement in massage or cryotherapy, and the consumption of alcohol, protein supplements, vitamin and mineral supplements, and ergogenic aids during the study period.

Upon arriving at the laboratory for the third visit (pre), a trained phlebotomist will collect a venous blood sample in three vacutainers; one containing a serum separator (8.5 mL); and two containing no reagent (10 mL) (28.5 mL total). Following blood collection, the participants' limb circumference of the thigh will be measured with a standard measuring tape and their limb range of motion will be assessed with a goniometer. Muscle soreness will be measured by use of a visual analogue scale (VAS) questionnaire while performing a standard bodyweight squat, and the pressure-pain threshold (PPT) test using a standard pressure algometer. Participants will then perform a single bout of resistance exercise at their allocated load intensity, firstly on the leg press and secondly, on the leg extension, separated by five-minutes rest. The exercise protocol will consist of a warm-up set (10 repetitions at 50% of the participants pre-established 1RM), followed by three sets performed to volitional failure (when a full repetition can no longer be completed) at either 30% or 80% 1RM. Two-minutes of rest will be awarded between each set of exercise and the participants will be verbally encouraged throughout.

Immediately following the exercise bout (post), another venous blood sample will be taken (3 x 8.5-10 mL) and the measures of limb circumference, range of motion, and muscle soreness will be repeated. Then, participants will be provided a dairy yoghurt containing 20 g of milk protein to stimulate muscle repair and growth. These four measures (excluding protein consumption) will be repeated during the morning of visits four (+24 h), five (+48 h), six (+72 h), and seven (+168 h). Also, during visits six and seven, the 1RM strength test will be repeated on the leg press and leg extension. Over the experimental period, a total of 171 mL of blood will be collected from each participant. This is the equivalent of ~34 teaspoons and is a common amount obtained during exercise physiology research. The blood samples will be used to analyse serum concentrations of creatine kinase, myoglobin (markers of muscle damage), C-reactive protein, and interleukin-6 (markers of inflammation).

OBJECTIVE 2/PART B: This study will employ a randomised parallel groups design, for which 22 healthy females aged 18-35 years will be recruited. Prior to the first laboratory visit, prospective participants will have the opportunity to ask questions and gain further information about the study via a video/phone call with the lead researcher. If willing to participate, participants will be asked to electronically sign a consent form. Participants will be asked to record their menstrual cycle and inform the lead researcher at the onset of menses – this information will be used to arrange the time of the baseline assessments, as all measures will be conducted during the late follicular phase of the menstrual cycle.

For the first visit, which acts as a familiarisation session, participants will arrive at the laboratory in the morning and be screened for eligibility with a standard health and readiness to exercise questionnaire. Next, participants will be familiarised with the exercise equipment and demonstrated correct form. Then, participants' maximal strength will be estimated though a one repetition maximum (1RM) test on the leg press and leg extension machines. Approximately three days later, participants will return to the laboratory and a baseline assessment of body composition will be conducted using dual energy X-ray absorptiometry (DXA). The 1RM test will be repeated on the leg press and leg extension machines for confirmation of maximal strength. Participants will then be randomly assigned to either a protein or placebo group. The protein group will be consuming a whole-food source of milk protein (dairy yoghurt) providing 20 g of milk protein and the placebo group will consume a carbohydrate-based food source that contains the

same amount of calories.

Visit three will occur three weeks later. In between visits two and three, participants will be asked to record their habitual diet and activity for three days, which will be used to assess any between-group differences. For each subsequent assessment day, participants will be prescribed a standardised breakfast that will contain either the protein or placebo product to consume at home 3 hours prior to attending the laboratory. Participants will consume one dose of their allocated supplement in the laboratory after exercise and be given a further two doses to consume at home interspaced by four hours. Therefore, 4 doses of the supplement will be consumed in each 24-hour period for four consecutive days, though on the final testing day, only one dose will be consumed with breakfast (17 doses total). Otherwise, participants will be instructed to consume their habitual diet, and during each laboratory visit a 24-hour dietary recall will be conducted to assess any changes and between-group differences in dietary intake. Participants will also be instructed to abstain from the use of non-steroidal anti-inflammatory drugs, strenuous exercise, engagement in massage or cryotherapy, and the consumption of alcohol, additional protein supplements, vitamin and mineral supplements, and ergogenic aids during the study period.

Upon arriving at the laboratory for the third visit (pre), a trained phlebotomist will collect a venous blood sample in three vacutainers; one containing a serum separator (8.5 mL); and two containing no reagent (10 mL) (28.5 mL total). Following blood collection, the participants' limb circumference of the thigh will be measured with a standard measuring tape and their limb range of motion will be assessed with a goniometer. Muscle soreness will be measured by use of a visual analogue scale (VAS) questionnaire while performing a standard bodyweight squat, and the pressure-pain threshold (PPT) test using a standard pressure algometer. Participants will then perform a single bout of resistance exercise, firstly on the leg press and secondly, on the leg extension, separated by five-minutes rest. The exercise protocol will consist of a warm-up set (10 repetitions at 50% of the participants pre-established 1RM), followed by three sets performed to volitional failure (when a full repetition can no longer be completed) at 80% 1RM. Two-minutes of rest will be awarded between each set of exercise and the participants will be verbally encouraged throughout.

Immediately following the exercise bout (post), another venous blood sample will be taken (3 x 8.5-10 mL) and the measures of limb circumference, range of motion, and muscle soreness will be repeated. Then, participants will consume their allocated dietary supplement (protein or placebo). These procedures will be repeated during the morning of visits four (+24 h), five (+48 h), six (+72 h), and seven (+168 h). Also, during visits six and seven, the 1RM strength test will be repeated on the leg press and leg extension. Over the experimental period, a total of 171 mL of blood will be collected from each participant. This is the equivalent of ~34 teaspoons and is a common amount obtained during exercise physiology research. The blood samples will be used to analyse serum concentrations of creatine kinase, myoglobin (markers of muscle damage), C-reactive protein, and interleukin-6 (markers of inflammation).

A14-1. In which aspects of the research process have you actively involved, or will you involve, patients, service users, and/or their carers, or members of the public?

- ☐ Design of the research
- ☐ Management of the research
- ☐ Undertaking the research
- ☐ Analysis of results
- ☐ Dissemination of findings
- ☑ None of the above

Give details of involvement, or if none please justify the absence of involvement.
The participants recruited for this study will be healthy, non-active males and females and the study does not involve the development of a clinical or public health intervention. The focus is on basic science and therefore the involvement of the public in the deign of the research is not required.

4. RISKS AND ETHICAL ISSUES

RESEARCH PARTICIPANTS

A17-1. Please list the principal inclusion criteria (list the most important, max 5000 characters).

Males and females (PART A)
Females-only (PART B)

All other inclusion criteria apply to PART A and PART B:
Aged 18-35 years
BMI 18.5 -25.0 kg/m2
Untrained in resistance exercise
No known chronic disease or current acute illness
No current or recent (past 3 months) musculoskeletal injury
No frequent use (2x per week for past month) of non-steroidal anti-inflammatory drugs and compliant to abstain from use during experimental period
No recent or current engagement in massage or cryotherapy and compliant to abstain from use during experimental period
No current use of protein or antioxidant supplements
Females will be eumenorrheic >12 months
Absence of pregnancy and breast-feeding

A17-2. Please list the principal exclusion criteria (list the most important, max 5000 characters).

<18 years or >35 years of age
Underweight
Overweight/obese
Resistance trained
Current or recent injury
Pregnancy or breast-feeding
Lactose intolerant
Unwilling to provide blood samples, perform resistance exercise, or abstain from use of NSAID's and protein supplementation (unless instructed as part of the research)
Unwilling to abstain from other forms of exercise during the experimental period

RESEARCH PROCEDURES, RISKS AND BENEFITS

A18. Give details of all non-clinical intervention(s) or procedure(s) that will be received by participants as part of the research protocol. *These include seeking consent, interviews, non-clinical observations and use of questionnaires.*

Please complete the columns for each intervention/procedure as follows:

1. Total number of interventions/procedures to be received by each participant as part of the research protocol.
2. If this intervention/procedure would be routinely given to participants as part of their care outside the research, how many of the total would be routine?
3. Average time taken per intervention/procedure (minutes, hours or days)
4. Details of who will conduct the intervention/procedure, and where it will take place.

Intervention or procedure	1	2	3	4
Obtaining informed consent	2	0	5 mins	Alice Pearson;<br>Human Performance Laboratory, Durham University
General demographic and health questionnaire	2	0	5 mins	Completed by the participant in the presence of Alice Pearson;<br>Human Performance Laboratory, Durham University
Readiness to exercise questionnaire	2	0	5 mins	Completed by the participant in the presence of Alice Pearson;<br>Human Performance Laboratory, Durham University
Habitual diet and activity record	2	0	3 days	Alice Pearson will give instruction on how the record should be completed and then the participant will complete the record at home
24-hour dietary recall	10	0	5 mins	Alice Pearson;<br>Human Performance Laboratory, Durham University
Measurement of maximal leg strength	8	0	45	Alice Pearson;

			mins	Human Performance Laboratory, Durham University
Measurement of muscle soreness by pressure algometry and visual analogue scale questionnaire	12	0	15 mins	Alice Pearson; Human Performance Laboratory, Durham University
Measurement of leg range of motion by goniometry	12	0	5 mins	Alice Pearson; Human Performance Laboratory, Durham University
Measurement of leg circumference by standard tape measure	12	0	5 mins	Alice Pearson; Human Performance Laboratory, Durham University
Completion of a leg-based resistance exercise protocol	2	0	30 mins	Alice Pearson; Human Performance Laboratory, Durham University
Ingestion of milk protein (yoghurt) or carbohydrate-based placebo (Part B only)	17	5	5 mins	Alice Pearson will provide the milk protein for the participants to consume either at the laboratory or at their home

A19. Give details of any clinical intervention(s) or procedure(s) to be received by participants as part of the research protocol. *These include uses of medicinal products or devices, other medical treatments or assessments, mental health interventions, imaging investigations and taking samples of human biological material. Include procedures which might be received as routine clinical care outside of the research.*

Please complete the columns for each intervention/procedure as follows:

1. Total number of interventions/procedures to be received by each participant as part of the research protocol.
2. If this intervention/procedure would be routinely given to participants as part of their care outside the research, how many of the total would be routine?
3. Average time taken per intervention/procedure (minutes, hours or days).
4. Details of who will conduct the intervention/procedure, and where it will take place.

Intervention or procedure	1	2	3	4
Assessment of body composition by dual energy X-ray absorptiometry<br><br>Note: 2 scans for part A and 1 scan for part B	3	0	20 mins	Alice Pearson; Truscott Imaging Suite, Durham University
Venous blood sampling by venepuncture (28.5 mL per sample; 342 mL total)<br><br>Note: 6 samples for part A (171 mL) and 6 samples for part B (171 mL) of the research	12	0	15 mins	Alice Pearson; Human Performance Laboratory, Durham University

A21. How long do you expect each participant to be in the study in total?

PART A: Seven laboratory visits (20-90 mins per visit) over a one-month period.

PART B: Seven laboratory visits (20-90 mins per visit) over a one-month period.

A22. What are the potential risks and burdens for research participants and how will you minimise them?

For all studies, describe any potential adverse effects, pain, discomfort, distress, intrusion, inconvenience or changes to lifestyle. Only describe risks or burdens that could occur as a result of participation in the research. Say what steps would be taken to minimise risks and burdens as far as possible.

The collection of blood samples may cause discomfort, minor bruising, and carries a risk of infection - blood samples will be collected by a trained phlebotomist wearing the correct personal protective equipment (e.g. disposable gloves, laboratory coat). All surfaces (e.g. chair, tables) will be cleaned before and after use and the researcher will practice hand washing prior to touching any equipment.

Performing the resistance exercise may cause musculoskeletal injury - A readiness to exercise questionnaire will be

completed prior to testing and participants will not be eligible to participate if they have a current or previous (past 3 months) musculoskeletal injury. Participants will be demonstrated correct lifting technique; perform an appropriate warm-up; and be supervised at all times.

Performing the Pressure-Pain Threshold test with a pressure algometer may cause discomfort and minor bruising - The participant will be instructed to indicate when the pressure applied with the algometer becomes painful, at which point, the pressure will be released. The test will be performed on alternate legs to reduce bruising of the same site and participants will be advised to treat any bruising with rest, ice, compression, and elevation.

Assessing body composition by dual energy X-ray absorptiometry exposes the participant to a small amount of ionising radiation. The effective dose has been reviewed and approved by the Medical Physics Expert and the Clinical Radiation Expert. Immediately prior to each DXA scan, female participants will be required to sign confirmation that they are not pregnant nor do they suspect that they may be pregnant.

There is a risk of spreading or being infected with Covid-19. The laboratory has a maximum occupancy that will be adhered to; a 2 metre distance will be maintained where possible; where close contact is required e.g. when taking blood samples, performing the pressure-pain threshold test, and measuring leg circumference, a face covering will be worn by both the researcher and participant and the researcher will wear disposable gloves; all surfaces including the exercise equipment will be cleaned before and after use with the correct cleaning solution; frequent hand-washing will be performed; and if either a participant or the researcher develops Covid-19 symptoms they will be instructed not to attend the laboratory and to self-isolate in line with government guidance.

A24. What is the potential for benefit to research participants?

Participants will receive a full body composition analysis from their DXA scans and a dietary analysis performed by a registered sport nutritionist. Participants will be informed of their maximum leg strength (1RM) which can be useful for exercise training programmes. Participants will also learn how to complete resistance exercises which can be of benefit to their musculoskeletal health.

RECRUITMENT AND INFORMED CONSENT

In this section we ask you to describe the recruitment procedures for the study. Please give separate details for different study groups where appropriate.

A27-1. How will potential participants, records or samples be identified? Who will carry this out and what resources will be used?*For example, identification may involve a disease register, computerised search of GP records, or review of medical records. Indicate whether this will be done by the direct healthcare team or by researchers acting under arrangements with the responsible care organisation(s).*

Participants will be identified from the Durham University staff and student base, as well as external to the University but locally to the region. Participants will be recruited by the primary researcher and supervisors. A recruitment email detailing the research project and eligibility criteria, including a participant information sheet, will be circulated around the university staff and student mailing list and via the weekly 'dialogue' newsletter. Posters detailing the study will be displayed in communal areas around the university, with contact details of the primary researcher. Social media (Twitter, LinkedIn) will be used to advertise the study, alongside details of how to get more information and to contact the primary researcher.

A27-2. Will the identification of potential participants involve reviewing or screening the identifiable personal information of patients, service users or any other person?

○ Yes ◉ No

Please give details below:

A28. Will any participants be recruited by publicity through posters, leaflets, adverts or websites?

◉ Yes ○ No

If Yes, please give details of how and where publicity will be conducted, and enclose copy of all advertising material (with version numbers and dates).

Posters will be placed in communal areas of Durham University buildings. An electronic version of the poster will be distributed via social media platforms (Twitter, LinkedIn). These will detail the study and provide contact details of the lead researcher.

A29. How and by whom will potential participants first be approached?

The participants will be recruited by the primary researcher and supervisors. A recruitment email detailing the research project and eligibility criteria, including a participant information sheet, will be circulated around the university staff and student mailing list. Interested participants will be asked to contact the lead researcher for further detail.

A30-1. Will you obtain informed consent from or on behalf of research participants?

(•) Yes ( ) No

If you will be obtaining consent from adult participants, please give details of who will take consent and how it will be done, with details of any steps to provide information (a written information sheet, videos, or interactive material). Arrangements for adults unable to consent for themselves should be described separately in Part B Section 6, and for children in Part B Section 7.

If you plan to seek informed consent from vulnerable groups, say how you will ensure that consent is voluntary and fully informed.

Participants will be provided a participant information sheet and privacy notice, alongside the informed consent form. They will be given the opportunity to ask any questions prior to signing the informed consent form, which will be received by the lead researcher.

If you are not obtaining consent, please explain why not.

Please enclose a copy of the information sheet(s) and consent form(s).

A30-2. Will you record informed consent (or advice from consultees) in writing?

(•) Yes ( ) No

A31. How long will you allow potential participants to decide whether or not to take part?

Participants will have as long as they wish to decide whether or not to participate, up until the study has terminated. The minimum time for consideration will be 7 days.

A32. Will you recruit any participants who are involved in current research or have recently been involved in any research prior to recruitment?

(•) Yes
( ) No
( ) Not Known

If Yes, please give details and justify their inclusion. If Not Known, what steps will you take to find out?

Participants who are currently taking part in another research project will not be included. Participants who have previously been involved in research may be included, but not if the research involved dietary and/or exercise intervention and was conducted in the past 6 months.

A33-1. What arrangements have been made for persons who might not adequately understand verbal explanations or written information given in English, or who have special communication needs?*(e.g. translation, use of interpreters)*

There are not the funds to support a translator and therefore individuals who are unable to understand the written or verbal information, will not be permitted to take part in the study.

A35. What steps would you take if a participant, who has given informed consent, loses capacity to consent during the study? *Tick one option only.*

- (•) The participant and all identifiable data or tissue collected would be withdrawn from the study. Data or tissue which is not identifiable to the research team may be retained.
- ( ) The participant would be withdrawn from the study. Identifiable data or tissue already collected with consent would be retained and used in the study. No further data or tissue would be collected or any other research procedures carried out on or in relation to the participant.
- ( ) The participant would continue to be included in the study.
- ( ) Not applicable – informed consent will not be sought from any participants in this research.
- ( ) Not applicable – it is not practicable for the research team to monitor capacity and continued capacity will be assumed.

Further details:

CONFIDENTIALITY

In this section, personal data means any data relating to a participant who could potentially be identified. It includes pseudonymised data capable of being linked to a participant through a unique code number.

Storage and use of personal data during the study

A36. Will you be undertaking any of the following activities at any stage (including in the identification of potential participants)?*(Tick as appropriate)*

- [ ] Access to medical records by those outside the direct healthcare team
- [ ] Access to social care records by those outside the direct social care team
- [x] Electronic transfer by magnetic or optical media, email or computer networks
- [ ] Sharing of personal data with other organisations
- [ ] Export of personal data outside the EEA
- [x] Use of personal addresses, postcodes, faxes, emails or telephone numbers
- [ ] Publication of direct quotations from respondents
- [ ] Publication of data that might allow identification of individuals
- [ ] Use of audio/visual recording devices
- [x] Storage of personal data on any of the following:
  - [x] Manual files (includes paper or film)
  - [ ] NHS computers
  - [ ] Social Care Service computers
  - [ ] Home or other personal computers
  - [x] University computers
  - [ ] Private company computers
  - [x] Laptop computers

Further details:
Personal information will only be used to contact the participant. All information will be stored on password protected devices and an encrypted memory stick.

A38. How will you ensure the confidentiality of personal data?*Please provide a general statement of the policy and procedures for ensuring confidentiality, e.g. anonymisation or pseudonymisation of data.*

All data files will be stored on computers or laptops with passwords that are only known by the research team and any hard copies will be stored in a locked filling cabinet which only the researchers can access.
Participants' names will not be used to label data files and samples collected. Instead, each will be given an anonymous participant code, which will only be known by the research team. This way none of the other participants or anyone outside of the research team will be able to identify which test data belongs to which participant.
The data presented in the written manuscript will not be identifiable to specific participants.
After six months, all data will be anonymised and identifiable original data will be destroyed.

A40. Who will have access to participants' personal data during the study? *Where access is by individuals outside the direct care team, please justify and say whether consent will be sought.*

Only the lead researcher (Alice Pearson) and supervisors (Dr Karen Hind and Dr Lindsay MacNaughton) will have access to participants' personal data.

Storage and use of data after the end of the study

A43. How long will personal data be stored or accessed after the study has ended?

- ( ) Less than 3 months
- (x) 3 – 6 months
- ( ) 6 – 12 months
- ( ) 12 months – 3 years
- ( ) Over 3 years

INCENTIVES AND PAYMENTS

A46. Will research participants receive any payments, reimbursement of expenses or any other benefits or incentives for taking part in this research?

( ) Yes (x) No

A47. Will individual researchers receive any personal payment over and above normal salary, or any other benefits or incentives, for taking part in this research?

( ) Yes (x) No

A48. Does the Chief Investigator or any other investigator/collaborator have any direct personal involvement (e.g. financial, share holding, personal relationship etc.) in the organisations sponsoring or funding the research that may give rise to a possible conflict of interest?

( ) Yes (x) No

NOTIFICATION OF OTHER PROFESSIONALS

A49-1. Will you inform the participants' General Practitioners (and/or any other health or care professional responsible for their care) that they are taking part in the study?

○ Yes ◉ No

If Yes, please enclose a copy of the information sheet/letter for the GP/health professional with a version number and date.

PUBLICATION AND DISSEMINATION

A50. Will the research be registered on a public database?

The UK Policy Framework for Health and Social Care Research sets out the principle of making information about research publicly available. Furthermore: Article 19 of the World Medical Association Declaration of Helsinki adopted in 2008 states that "every clinical trial must be registered on a publicly accessible database before recruitment of the first subject"; and the International Committee of Medical Journal Editors (ICMJE) will consider a clinical trial for publication only if it has been registered in an appropriate registry. Please see guidance for more information.

◉ Yes ○ No

Please give details, or justify if not registering the research.
The study will be registered on a clinical trials registry following ethical approval - https://www.isrctn.com/

Please ensure that you have entered registry reference number(s) in question A5-1.

A51. How do you intend to report and disseminate the results of the study? *Tick as appropriate:*

- [x] Peer reviewed scientific journals
- [ ] Internal report
- [x] Conference presentation
- [ ] Publication on website
- [ ] Other publication
- [ ] Submission to regulatory authorities
- [ ] Access to raw data and right to publish freely by all investigators in study or by Independent Steering Committee on behalf of all investigators
- [ ] No plans to report or disseminate the results
- [x] Other (please specify)

PhD Thesis

A53. How and when will you inform participants of the study results?

If there will be no arrangements in place to inform participants please justify this.
Participants will be offered a lay summary via email, once the results have been published in a peer review journal.

5. Scientific and Statistical Review

A54. How has the scientific quality of the research been assessed? *Tick as appropriate:*

- [ ] Independent external review
- [ ] Review within a company
- [ ] Review within a multi−centre research group
- [ ] Review within the Chief Investigator's institution or host organisation
- [x] Review within the research team
- [x] Review by educational supervisor
- [ ] Other

Justify and describe the review process and outcome. If the review has been undertaken but not seen by the researcher, give details of the body which has undertaken the review:
The study proposal has been reviewed by Dr Karen Hind and Dr Lindsay MacNaughton. This review included concept and supporting literature, objectives, study design, inclusion/exclusion criteria, recruitment of participants, primary and secondary outcome measures, methodology and the statistical analysis plan. In addition, the proposal has been reviewed by a panel of independent academics at Durham University, as part of the annual doctorate review. The review process has taken approximately 3 months and the proposal was approved by the supervisory team.

For all studies except non-doctoral student research, please enclose a copy of any available scientific critique reports, together with any related correspondence.

For non-doctoral student research, please enclose a copy of the assessment from your educational supervisor/ institution.

A56. How have the statistical aspects of the research been reviewed? *Tick as appropriate:*

- [ ] Review by independent statistician commissioned by funder or sponsor
- [ ] Other review by independent statistician
- [ ] Review by company statistician
- [ ] Review by a statistician within the Chief Investigator's institution
- [ ] Review by a statistician within the research team or multi−centre group
- [x] Review by educational supervisor
- [ ] Other review by individual with relevant statistical expertise
- [ ] No review necessary as only frequencies and associations will be assessed – details of statistical input not required

In all cases please give details below of the individual responsible for reviewing the statistical aspects. If advice has been provided in confidence, give details of the department and institution concerned.

	Title	Forename/Initials	Surname
	Dr	Karen	Hind
Department	Sport and Exercise Sciences		
Institution	Durham University		
Work Address	42 Old Elvet		
	Durham		
Post Code	DH1 3HN		
Telephone			
Fax			
Mobile			
E-mail	karen.hind@durham.ac.uk		

Please enclose a copy of any available comments or reports from a statistician.

A57. What is the primary outcome measure for the study?

Exercise-induced changes in markers of muscle damage

A58. What are the secondary outcome measures? *(if any)*

Precision assessment of dual energy X-ray absorptiometry

A59. What is the sample size for the research? *How many participants/samples/data records do you plan to study in*

total? If there is more than one group, please give further details below.

Total UK sample size: 62
Total international sample size (including UK):
Total in European Economic Area:

Further details:
PART A: 20 males and 20 females

PART B: 22 females

A60. How was the sample size decided upon? *If a formal sample size calculation was used, indicate how this was done, giving sufficient information to justify and reproduce the calculation.*

PART A: A statistical power analysis was conducted. It was determined that a sample size of 8 per group was required to have 80% power to detect a between-group difference in muscle strength of 14%, when using a dependent t-test with a 0.05 two-sided significance level. A ~20% drop-out is anticipated, therefore, 10 males and 10 females in both groups will be recruited (n = 40 total).

PART B: A statistical power analysis was conducted. It was determined that a sample size of 9 per group was required to have 80% power to detect a between-group difference in muscle strength of 13%, when using a dependent t-test with a 0.05 two-sided significance level. A ~20% drop-out is anticipated, therefore, a total of 22 females will be recruited.

A61. Will participants be allocated to groups at random?

(•) Yes ( ) No

If yes, please give details of the intended method of randomisation:
With each eligible participant identified, a coin will be tossed to decide which group they go into.

A62. Please describe the methods of analysis (statistical or other appropriate methods, e.g. for qualitative research) by which the data will be evaluated to meet the study objectives.

PART A: The Shapiro Wilk test will be used to assess the distribution of the data and to ensure no assumptions for statistical models are violated. Any data that violate the assumptions will be logarithmically transformed before statistical analysis. A two-way mixed design analysis of variance (ANOVA) will be used to analyse each muscle damage marker between exercise conditions and within time points. A two-way between-subjects ANOVA will be used to analyse each muscle damage marker between exercise conditions and between sexes.
Independent t tests with Bonferroni corrections will be used to compare time-points between exercise conditions for all measures. Paired t tests will be used to detect any significant within-group changes between time points.

PART B: The Shapiro Wilk test will be used to assess the distribution of the data and to ensure no assumptions for statistical models are violated. Any data that violate the assumptions will be logarithmically transformed before statistical analysis. A two-way mixed design ANOVA will be used to analyse each muscle damage marker between dietary conditions (protein or placebo) and within time points. Independent t tests with Bonferroni corrections will be used to compare time-points between dietary conditions for all measures. Paired t tests will be used to detect any significant within-group changes between time points.

6. MANAGEMENT OF THE RESEARCH

A63. Other key investigators/collaborators. *Please include all grant co−applicants, protocol co−authors and other key members of the Chief Investigator's team, including non-doctoral student researchers.*

Title	Forename/Initials	Surname
Dr	Karen	Hind

Post	Assistant Professor
Qualifications	PhD
Employer	Durham University
Work Address	42 Old Elvet
	Durham
Post Code	DH1 3HN
Telephone	
Fax	
Mobile	
Work Email	karen.hind@durham.ac.uk

Title	Forename/Initials	Surname
Dr	Lindsay	MacNaughton

Post	Assistant Professor
Qualifications	PhD, SENr
Employer	Durham University
Work Address	42 Old Elvet
	Durham
Post Code	DH1 3HN
Telephone	
Fax	
Mobile	
Work Email	lindsay.s.macnaughton@durham.ac.uk

A64. Details of research sponsor(s)

A64-1. Sponsor

Lead Sponsor

Status:
- ○ NHS or HSC care organisation
- ● Academic
- ○ Pharmaceutical industry
- ○ Medical device industry
- ○ Local Authority
- ○ Other social care provider (including voluntary sector or private organisation)
- ○ Other

If Other, please specify:

Commercial status: Non-Commercial

Contact person

Name of organisation	Durham University
Given name	Niall

Family name O'Loughlin
Address
Town/city Durham
Post code
Country United Kingdom
Telephone
Fax
E-mail niall.c.o'loughlin@durham.ac.uk

A65. Has external funding for the research been secured?

Please tick at least one check box.

- [ ] Funding secured from one or more funders
- [ ] External funding application to one or more funders in progress
- [x] No application for external funding will be made

What type of research project is this?

- ( ) Standalone project
- ( ) Project that is part of a programme grant
- ( ) Project that is part of a Centre grant
- ( ) Project that is part of a fellowship/ personal award/ research training award
- (•) Other

Other – please state:
Doctoral research degree

A67. Has this or a similar application been previously rejected by a Research Ethics Committee in the UK or another country?

( ) Yes (•) No

Please provide a copy of the unfavourable opinion letter(s). You should explain in your answer to question A6-2 how the reasons for the unfavourable opinion have been addressed in this application.

A69-1. How long do you expect the study to last in the UK?

Planned start date: 01/05/2021
Planned end date: 01/05/2023
Total duration:

Years: 2 Months: 0 Days: 0

A70. Definition of the end of trial, and justification in the case where it is not the last visit of the last subject undergoing the trial (1)

When the final participant has completed their last trial.

A71-2. Where will the research take place? *(Tick as appropriate)*

- [x] England
- [ ] Scotland
- [ ] Wales
- [ ] Northern Ireland
- [ ] Other countries in European Economic Area

Total UK sites in study 1

Does this trial involve countries outside the EU?
( ) Yes (•) No

A72. Which organisations in the UK will host the research? *Please indicate the type of organisation by ticking the box and give approximate numbers if known:*

[ ] NHS organisations in England	
[ ] NHS organisations in Wales	
[ ] NHS organisations in Scotland	
[ ] HSC organisations in Northern Ireland	
[ ] GP practices in England	
[ ] GP practices in Wales	
[ ] GP practices in Scotland	
[ ] GP practices in Northern Ireland	
[ ] Joint health and social care agencies (eg community mental health teams)	
[ ] Local authorities	
[ ] Phase 1 trial units	
[ ] Prison establishments	
[ ] Probation areas	
[ ] Independent (private or voluntary sector) organisations	
[x] Educational establishments	1
[ ] Independent research units	
[ ] Other (give details)	
Total UK sites in study:	1

A76. Insurance/ indemnity to meet potential legal liabilities

Note: ***in this question to NHS indemnity schemes include equivalent schemes provided by Health and Social Care (HSC) in Northern Ireland***

A76-1. What arrangements will be made for insurance and/or indemnity to meet the potential legal liability of the sponsor(s) for harm to participants arising from the management of the research? *Please tick box(es) as applicable.*

Note: Where a NHS organisation has agreed to act as sponsor or co-sponsor, indemnity is provided through NHS schemes. Indicate if this applies (there is no need to provide documentary evidence). For all other sponsors, please describe the arrangements and provide evidence.

☐ NHS indemnity scheme will apply (NHS sponsors only)

☑ Other insurance or indemnity arrangements will apply (give details below)

University insurance (certificate attached)

Please enclose a copy of relevant documents.

A76-2. What arrangements will be made for insurance and/ or indemnity to meet the potential legal liability of the sponsor(s) or employer(s) for harm to participants arising from the design of the research? *Please tick box(es) as applicable.*

Note: Where researchers with substantive NHS employment contracts have designed the research, indemnity is provided through NHS schemes. Indicate if this applies (there is no need to provide documentary evidence). For other protocol authors (e.g. company employees, university members), please describe the arrangements and provide evidence.

☐ NHS indemnity scheme will apply (protocol authors with NHS contracts only)

☑ Other insurance or indemnity arrangements will apply (give details below)

University insurance (certificate attached)

Please enclose a copy of relevant documents.

A76-3. What arrangements will be made for insurance and/ or indemnity to meet the potential legal liability of investigators/collaborators arising from harm to participants in the conduct of the research?

Note: Where the participants are NHS patients, indemnity is provided through the NHS schemes or through professional indemnity. Indicate if this applies to the whole study (there is no need to provide documentary evidence). Where non-NHS sites are to be included in the research, including private practices, please describe the arrangements which will be made at these sites and provide evidence.

☐ NHS indemnity scheme or professional indemnity will apply (participants recruited at NHS sites only)

☑ Research includes non-NHS sites (give details of insurance/ indemnity arrangements for these sites below)

University insurance (certificate attached)

Please enclose a copy of relevant documents.

PART B: Section 3 – Exposure to ionising radiation

Complete sub-sections A and/or B as applicable with input from relevant experts. It is advisable to discuss the proposed research at an early stage with (a) a Medical Physics Expert and (b) a Clinical Radiation Expert, who will carry out the required assessments for sub-sections C and D. The lead MPE can also facilitate the completion of sub-sections A and/or B if necessary.

1. Does the study involve exposure to radioactive materials?

○ Yes ◉ No

To update the response above, go to the Project Filter Question 2 'Does the study involve exposure to radioactive materials?' and select an option.

2. Does the study involve other diagnostic or therapeutic ionising radiation?

◉ Yes ○ No

A. Radioactive materials

Details of radioactive materials

B. Other ionising radiation

B1. Details of other ionising radiation

Give details by completing the table below:

Procedure	No of procedures	Estimated procedure dose *(use national Diagnostic Reference Levels where available)*
Dual energy X-ray absorptiometry total body scan	PART A: 2 scans<br><br>PART B: 1 scan	0.002 mSv

C. Dose and risk assessment

C1. What is the total participant dose from all the exposures in A1 and/or B1, and what component of this is the additional dose over and above standard practice? What are the risks associated with these two doses (total and additional)?

The dose and risk assessment should be set out below. This should be prepared by a Medical Physics Expert (MPE) who is a registered clinical scientist registered with the Health Professions Council and has expertise relevant to the planned exposures. Where the study involves different types of exposure (for example, both radioactive materials and other ionising radiation, or more than one imaging method), advice may need to be sought from other MPEs with relevant expertise. The lead MPE should produce a combined assessment for the ethics committee, giving the names of any other MPEs who have contributed to the assessment. Further guidance is available by clicking on the information button.

This study requires exposure to ionising radiation which is detailed in B1. All exposures required by the study are additional to routine clinical care. The total procedure dose is 0.004mSv (Part A) or 0.002mSv (Part B). This is equivalent to an average natural background radiation in the UK for a period of 13 hours and 6.5 hours respectively.

Ionising radiation can cause cancer which manifests itself after many years or decades. The risk of developing cancer as a consequence of taking part in this study is 0.00002% and 0.00001% respectively, which is low. For comparison, the natural lifetime cancer incidence in the general population is 50%.

This assessment was produced by David Rawlings who is not part of the trial team.

Special attention must be paid to pregnant/potentially pregnant women or those who are breast feeding, or other potentially vulnerable groups.

C2. Declaration by lead Medical Physics Expert

I am satisfied that the information in sub-sections A and/or B and the assessment in sub-section C provide a reasonable estimate of the ionising radiation exposure planned in this research and the associated risks.

This section was signed electronically by Mr David Rawlings on 08/03/2021 17:13.

Job Title/Post:	Clinical Scientist - Bank
Organisation:	Northern Medical Physics and Clinical Engineering
Email:	David.Rawlings1@nhs.net

C3. Details of person acting as lead Medical Physics Expert

	Title	Forename/Initials	Surname
	Mr	David	Rawlings

Post: Clinical Scientist - Bank

Details of clinical scientist registration with the Health Professions Council:

Registration no: CS01872

Organisation: Newcastle upon Tyne Hospitals NHS Foundation Trust

Address: Northern Medical Physics and Clinical Engineering
Freeman Hospital

Post Code: NE77DN

Telephone: 07973243309

Fax:

Mobile: 07973243309

Email: david.rawlings1@nhs.net

D. Clinical assessment

This sub-section should be completed by a Clinical Radiation Expert (CRE) who is a registered doctor or dentist with clinical expertise relevant to the planned exposures. The assessment should cover potential exposure at all research sites, taking account of possible variation in normal clinical practice. Where the study involves different types of exposure (for example, both radiotherapy and other ionising radiation), advice may need to be sought from other CREs with relevant expertise. The lead CRE should produce a combined assessment for the ethics committee, giving the names of any other CREs who have contributed to the assessment. The guidance notes give advice to Chief Investigators on who can act as lead Clinical Radiation Expert (CRE) and advice for the CRE on the assessment of exposures having regard to IRMER.

Special attention must be paid to pregnant/potentially pregnant women or those who are breast feeding, or other potentially vulnerable groups.

D1. Will the exposure exceed the exposure that might be received as part of normal care at any proposed research site?

(●) Yes ( ) No

D2. Assessment of additional exposure

Explain how the planned exposure compares with normal practice and assess whether it is appropriate, using language comprehensible to a lay person. Consideration should be given to the specific objectives of the exposure, the characteristics of participants, the potential diagnostic or therapeutic benefits to the participant, the potential benefits to society, the risk to the participant and the availability of alternative techniques involving less, or no, ionising radiation.

If pregnant or breast-feeding mothers are to be studied give reasons and details of special radiation protection measures to be taken.

This study involves ionising radiation in the form of DXA scan. The radiation dose that participants in this study will receive has been calculated as equivalent to less than one day of background radiation. Pregnant and breast-feeding women will not be included in the study. Only DXA can provide the accurate quantification of lean mass that is required for this study, which will have significant benefits to both the individual participant and this field of research in terms of understanding muscle damage with low and higher intensity resistance exercise training in men and women and the potential for milk-based protein to reduce muscle damage in women. This can then be translated into knowledge and practice to the benefit of supporting health. Therefore, the benefits of this study are far greater than the small risk associated with the risk of using low dose ionising radiation.

D3. Declaration by lead Clinical Radiation Expert

I am satisfied that the exposure to ionising radiation planned in this research study (as defined in A1 and/or B1) is

reasonable and that the risks are adequately described in the participant information sheet for the study.

This section was signed electronically by Dr Nicola Keay on 25/02/2021 09:28.

Job Title/Post:	Honorary Fellow
Organisation:	Durham University
Email:	nickykeayfrancis@googlemail.com

D4. Details of lead Clinical Radiation Expert

	Title Forename/Initials Surname<br>Dr Nicola Keay
Post	Honorary Fellow, Sport and Exercise Sciences, Durham University
Details of professional registration	(●) General Medical Council ( ) General Dental Council
Registration no	3466230
Organisation	Sport and Exercise Sciences, Durham University
Address	42 Old Elvet
Post Code	DH1 3HN
Telephone	
Fax	
Mobile	
Email	nickykeayfrancis@googlemail.com

Employers responsible for radiation facilities at research sites must have written procedures to meet the requirements of the Ionising Radiation (Medical Exposure) Regulations 2000 (IRMER). R & D offices for NHS sites will seek confirmation from local radiation experts that local IRMER authorisation procedures have been followed. Where the local Medical Physics Expert or IRMER Practitioner disagrees with the assessments made in this Section and/or the care organisation is unable to adhere to the protocol, this should be discussed with the Chief Investigator and the lead experts for the study. Any necessary variation in the protocol or participant information sheet at particular sites should be notified to the main REC as a substantial amendment and an ethical opinion sought.

Part B: Section 5 – Use of newly obtained human tissue(or other human biological materials) for research purposes

1. What types of human tissue or other biological material will be included in the study?

Blood samples will be collected, from which only the serum will be analysed. No relevant material under the HTA will be analysed or stored (plasma will be destroyed).

2. Who will collect the samples?

The lead researcher is trained in phlebotomy and will collect the blood samples.

3. Who will the samples be removed from?

- [x] Living donors
- [ ] The deceased

4. Will informed consent be obtained from living donors for use of the samples? *Please tick as appropriate*

In this research?

(•) Yes ( ) No

In future research?

( ) Yes ( ) No (•) Not applicable

6. Will any tissues or cells be used for human application or to carry out testing for human application in this research?

( ) Yes (•) No

8. Will the samples be stored: *[Tick as appropriate]*

In fully anonymised form? *(link to donor broken)*

(•) Yes ( ) No

In linked anonymised form? *(linked to stored tissue but donor not identifiable to researchers)*

( ) Yes (•) No

In a form in which the donor could be identifiable to researchers?

( ) Yes (•) No

9. What types of test or analysis will be carried out on the samples?

The blood samples will undergo centrifugation to separate the serum, which will then be frozen, and the remaining blood particles will be disposed. The serum samples will be analysed by an enzyme-linked immunosorbent assay to determine concentrations of creatine kinase, myoglobin, C-reactive protein, and interleukin-6.

10. Will the research involve the analysis or use of human DNA in the samples?

( ) Yes (•) No

11. Is it possible that the research could produce findings of clinical significance for donors or their relatives?

○ Yes ● No

12. If so, will arrangements be made to notify the individuals concerned?

○ Yes ○ No ● Not applicable

13. Give details of where the samples will be stored, who will have access and the custodial arrangements.

The samples will be stored in a locked -80 degrees Celsius freezer situated in the Human Performance Laboratory at the Sports and Wellbeing Park, Durham University.
Only the research team will have access to the samples.

14. What will happen to the samples at the end of the research? *Please tick all that apply and give further details.*

☐ Transfer to research tissue bank

(If the bank is in England, Wales or Northern Ireland the institution will require a licence from the Human Tissue Authority to store relevant material for possible further research.)

☐ Storage by research team pending ethical approval for use in another project

(Unless the researcher's institution holds a storage licence from the Human Tissue Authority, or the tissue is stored in Scotland, or it is not relevant material, a further application for ethical review should be submitted before the end of this project.)

☐ Storage by research team as part of a new research tissue bank

(The institution will require a licence from the Human Tissue Authority if the bank will be storing relevant material in England, Wales or Northern Ireland. A separate application for ethical review of the tissue bank may also be submitted.)

☑ Storage by research team of biological material which is not "relevant material" for the purposes of the Human Tissue Act

☐ Disposal in accordance with the Human Tissue Authority's Code of Practice

☐ Other

☐ Not yet known

Please give further details of the proposed arrangements:

The samples will be stored (fully anonymised) for potential future analysis as part of new research and will be disposed of after 5 years.

PART C: Overview of research sites

Please enter details of the host organisations (Local Authority, NHS or other) in the UK that will be responsible for the research sites. *For further information please refer to guidance.*

Research site		Investigator/ Collaborator/ Contact	
Institution name	Durham University	Title	Miss
Department name	Sport and Exercise Sciences	First name/ Initials	Alice
Street address	Sports and Wellbeing Park, Durham University, Maiden Castle, Stockton Road	Surname	Pearson
Town/city	Durham		
Post Code	DH1 3SE		

PART D: Declarations

D1. Declaration by Chief Investigator

1. The information in this form is accurate to the best of my knowledge and belief and I take full responsibility for it.
2. I undertake to fulfil the responsibilities of the chief investigator for this study as set out in the UK Policy Framework for Health and Social Care Research.
3. I undertake to abide by the ethical principles underlying the Declaration of Helsinki and good practice guidelines on the proper conduct of research.
4. If the research is approved I undertake to adhere to the study protocol, the terms of the full application as approved and any conditions set out by review bodies in giving approval.
5. I undertake to notify review bodies of substantial amendments to the protocol or the terms of the approved application, and to seek a favourable opinion from the main REC before implementing the amendment.
6. I undertake to submit annual progress reports setting out the progress of the research, as required by review bodies.
7. I am aware of my responsibility to be up to date and comply with the requirements of the law and relevant guidelines relating to security and confidentiality of patient or other personal data, including the need to register when necessary with the appropriate Data Protection Officer. I understand that I am not permitted to disclose identifiable data to third parties unless the disclosure has the consent of the data subject or, in the case of patient data in England and Wales, the disclosure is covered by the terms of an approval under Section 251 of the NHS Act 2006.
8. I understand that research records/data may be subject to inspection by review bodies for audit purposes if required.
9. I understand that any personal data in this application will be held by review bodies and their operational managers and that this will be managed according to the principles established in the Data Protection Act 2018.
10. I understand that the information contained in this application, any supporting documentation and all correspondence with review bodies or their operational managers relating to the application:
    - Will be held by the REC (where applicable) until at least 3 years after the end of the study; and by NHS R&D offices (where the research requires NHS management permission) in accordance with the NHS Code of Practice on Records Management.
    - May be disclosed to the operational managers of review bodies, or the appointing authority for the REC (where applicable), in order to check that the application has been processed correctly or to investigate any complaint.
    - May be seen by auditors appointed to undertake accreditation of RECs (where applicable).
    - Will be subject to the provisions of the Freedom of Information Acts and may be disclosed in response to requests made under the Acts except where statutory exemptions apply.
    - May be sent by email to REC members.
11. I understand that information relating to this research, including the contact details on this application, may be held on national research information systems, and that this will be managed according to the principles established in the Data Protection Act 2018.
12. Where the research is reviewed by a REC within the UK Health Departments Research Ethics Service, I understand that the summary of this study will be published on the website of the Health Research Authority (HRA) together with the contact point for enquiries named below. Publication will take place no earlier than 3 months after the issue of the ethics committee’s final opinion or the withdrawal of the application.

Contact point for publication*(Not applicable for R&D Forms)*

HRA would like to include a contact point with the published summary of the study for those wishing to seek further

information. We would be grateful if you would indicate one of the contact points below.

- Chief Investigator
- Sponsor
- Study co-ordinator
- Student
- Other – please give details
- None

Access to application for training purposes *(Not applicable for R&D Forms)*

Optional – please tick as appropriate:

☐ I would be content for members of other RECs to have access to the information in the application in confidence for training purposes. All personal identifiers and references to sponsors, funders and research units would be removed.

This section was signed electronically by Miss Alice Pearson on 04/05/2021 11:05.

Job Title/Post:	PhD Researcher
Organisation:	Durham University
Email:	

D2. Declaration by the sponsor's representative

If there is more than one sponsor, this declaration should be signed on behalf of the co−sponsors by a representative of the lead sponsor named at A64-1.

I confirm that:

1. This research proposal has been discussed with the Chief Investigator and agreement in principle to sponsor the research is in place.

2. An appropriate process of scientific critique has demonstrated that this research proposal is worthwhile and of high scientific quality.

3. Any necessary indemnity or insurance arrangements, as described in question A76, will be in place before this research starts. Insurance or indemnity policies will be renewed for the duration of the study where necessary.

4. Arrangements will be in place before the study starts for the research team to access resources and support to deliver the research as proposed.

5. Arrangements to allocate responsibilities for the management, monitoring and reporting of the research will be in place before the research starts.

   Please note: *The declarations below do not form part of the application for approval above. They will not be considered by the Research Ethics Committee.*

6. Where the research is reviewed by a REC within the UK Health Departments Research Ethics Service, I understand that the summary of this study will be published on the website of the National Research Ethics Service (NRES), together with the contact point for enquiries named in this application. Publication will take place no earlier than 3 months after issue of the ethics committee's final opinion or the withdrawal of the application.

7. Specifically, for submissions to the Research Ethics Committees (RECs) I declare that any and all clinical trials approved by the HRA since 30th September 2013 (as defined on IRAS categories as clinical trials of medicines, devices, combination of medicines and devices or other clinical trials) have been registered on a publically accessible register in compliance with the HRA registration requirements for the UK, or that any deferral granted by the HRA still applies.

This section was signed electronically by Mr Niall C O'Loughlin on 07/05/2021 09:12.

Job Title/Post:	Jane Macnaughton, Professor of Medical Humanities
Organisation:	Durham University
Email:	research.policy@durham.ac.uk

D3. Declaration for student projects by academic supervisor(s)

1. I have read and approved both the research proposal and this application. I am satisfied that the scientific content of the research is satisfactory for an educational qualification at this level.

2. I undertake to fulfil the responsibilities of the supervisor for this study as set out in the UK Policy Framework for Health and Social Care Research.

3. I take responsibility for ensuring that this study is conducted in accordance with the ethical principles underlying the Declaration of Helsinki and good practice guidelines on the proper conduct of research, in conjunction with clinical supervisors as appropriate.

4. I take responsibility for ensuring that the applicant is up to date and complies with the requirements of the law and relevant guidelines relating to security and confidentiality of patient and other personal data, in conjunction with clinical supervisors as appropriate.

Academic supervisor 1

This section was signed electronically by Dr Lindsay Macnaughton on 04/05/2021 14:27.

Job Title/Post:	Assistant Professor
Organisation:	Durham University
Email:	lindsay.s.macnaughton@durham.ac.uk

Academic supervisor 2

This section was signed electronically by Dr Karen Hind on 04/05/2021 14:26.

Job Title/Post:	Assistant Professor
Organisation:	Durham University
Email:	karen.hind@durham.ac.uk
