# Supplementary material for: Sex differences in the impact of resistance exercise load on muscle damage: A protocol for a randomised parallel group trial
Source: PLoS One. 2022 Sep 29;17(9):e0275221. doi: 10.1371/journal.pone.0275221 (PMC9521925; doi:10.1371/journal.pone.0275221)
Supplement: S1 Checklist — (DOC) [file pone.0275221.s003.doc]

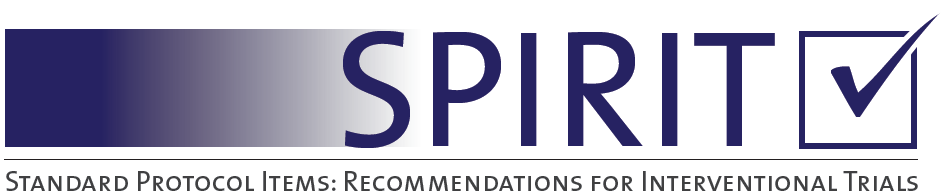


SPIRIT 2013 Checklist: Recommended items to address in a clinical trial protocol and related documents*

| Section/item | ItemNo | Description |
| --- | --- | --- |
| **Administrative information** | | |
| Title | 1 | Impact of resistance exercise load on muscle damage in untrained males and females: A protocol for a randomised parallel group trial (EIMD-LOAD) |
| Trial registration | 2a | This study has been registered at ClinicalTrials.gov (ID: NCT05111054). |
| Protocol version | 3 | 10/11/2021 V1 |
| Funding | 4 | This work received no specific funding. |
| Roles and responsibilities | 5a | Alice G. Pearson^1^ (PhD Researcher), Lindsay S. Macnaughton^1^ (Assistant Professor), Karen Hind^1^ (Associate Professor)  ^1^Department of Sport and Exercise Sciences, Durham University, Durham, United Kingdom |
| Introduction |  |  |
| Background and rationale | 6a | Unaccustomed resistance exercise can cause muscle damage, presenting as muscle soreness and reduced muscle function - such as loss of strength, power, and flexibility - for several days after the exercise bout. Therefore, individuals may require longer recovery periods before performing another exercise bout, and their performance may be impaired. Further, muscle soreness may reduce exercise compliance, particularly in novice individuals. Over time, this may compromise the gains in muscle mass and strength achieved through exercise training. Therefore, strategies to reduce the severity of exercise-induced muscle damage and/or to enhance post-exercise recovery processes are advantageous for exercising individuals.  One such strategy is to perform resistance exercise with lighter loads, i.e., <70% one repetition maximum (1RM). Low-load resistance training has shown to induce comparable gains in muscle mass and strength to high-load (≥70% 1RM), while being perceptively less exerting. Low-load resistance exercise may place less mechanical stress on muscle fibres and accordingly, its impact on muscle damage has been investigated. While several studies have reported less severe muscle damage, muscle soreness, and functional impairments with low-load resistance exercise compared to high-load, others have found no differences. Further, there is a lack of studies conducted solely in females or comparing between sexes. It has been suggested that males and females respond differently to muscle damage, and therefore, this research aims to provide a sex comparison in the muscle damage response to an acute bout of resistance exercise performed with low or high loads. |
| Objectives | 7 | Aim: To provide a sex comparison in the muscle damage response to an acute bout of resistance exercise performed with low or high loads. |
|  |  | Hypothesis: It is expected that the resistance exercise protocol will induce muscle damage, which will be less severe in the low-load exercise condition. It cannot be ascertained whether males and females will have the same responses to the exercise. |
| Trial design | 8 | Randomised parallel group design with equivalent group allocation, stratified by sex. |
| Methods: Participants, interventions, and outcomes | | |
| Study setting | 9 | The study will take place in the Human Performance Laboratory and Truscott Imaging Suite at The Graham Sports Centre, Maiden Castle, Durham University, Durham, England. |
| Eligibility criteria | 10 | Inclusion Criteria:   - BMI 18.5 - 25.0 kg/m2 - Untrained in resistance exercise - No known chronic disease or current acute illness - No current or recent (past 3 months) musculoskeletal injury - No frequent use (2x per week for past month) of non-steroidal anti-inflammatory drugs, anti-oxidant supplements, polyunsaturated omega-3 fatty acids (and other substances that may alleviate muscle damage) and compliant to abstain from use during experimental period - No recent or current engagement in massage or cryotherapy and compliant to abstain from use during experimental period - Females will be eumenorrheic (regular menstrual cycle) >12 months - Absence of pregnancy and breast-feeding   Exclusion Criteria:   - Underweight - Overweight/obese - Resistance trained - Current or recent injury - Pregnancy or breast-feeding - Unwilling to provide blood samples, perform resistance exercise, or abstain from use of NSAID's and other substances (stated above) - Unwilling to abstain from other forms of exercise during the experimental period |
| Interventions | 11a | ‘Low-Load’ – Leg-based resistance exercise performed at 30% of the individual’s pre-determined one-repetition maximum (1RM). Firstly, a warm-up set of 10 repetitions will be performed at 50% 1RM, followed by 3 sets at 30% 1RM performed to volitional failure. Two minutes of rest will occur between each set. This protocol will be performed first on the leg extension machine, and second, on the leg curl machine separated by a rest period of 5 minutes.  ‘High-Load’ – Leg-based resistance exercise performed at 80% of the individual’s pre-determined one-repetition maximum (1RM). Firstly, a warm-up set of 10 repetitions will be performed at 50% 1RM, followed by 3 sets at 80% 1RM performed to volitional failure. Two minutes of rest will occur between each set. This protocol will be performed first on the leg extension machine, and second, on the leg curl machine separated by a rest period of 5 minutes.  This exercise protocol will be completed 3 wk after the baseline assessments. |
|  | 11d | Participants will be required to abstain from the following during the intervention period:   - Use non-steroidal anti-inflammatory drugs (e.g., ibuprofen) - Perform strenuous exercise (daily activity such as walking is permitted) - Engage in massage, stretching, or cryotherapy - Consume protein supplements, vitamin or mineral supplements, or ergogenic aids (e.g., creatine, pre-workout stimulants, steroidal drugs) - Consume alcohol (for the 48 hours prior to each visit) |
| Outcomes | 12 | 1) **Maximal Voluntary Contraction**: One-repetition maximum (1RM) test performed on leg extension and leg curl machines.  Time points measured: Baseline (-28 d pre-exercise), baseline (-25 d pre-exercise), 72 h post-exercise, 168 h post-exercise.  Analysis metric (mean ± standard deviation): Change from baseline (highest value) to 72 h post-exercise, change from baseline to 168 h post-exercise.  2) **Serum creatine kinase concentration** from venous blood sampling.  Time points measured: Immediately pre-exercise, immediately post-exercise, 24 h post-exercise, 48 h post-exercise,72 h post-exercise, 168 h post-exercise.  Analysis metric (mean ± standard deviation): Change from pre-exercise to immediately post-exercise, change from pre-exercise to 24 h to post-exercise, change from pre-exercise to 48 h to post-exercise, change from pre-exercise to 72 h post-exercise, change from pre-exercise to 168 h post-exercise.  3) **Serum interleukin-6 concentration** from venous blood sampling.  Time points measured: Immediately pre-exercise, immediately post-exercise, 24 h post-exercise, 48 h post-exercise,72 h post-exercise, 168 h post-exercise.  Analysis metric (mean ± standard deviation): Change from pre-exercise to immediately post-exercise, change from pre-exercise to 24 h to post-exercise, change from pre-exercise to 48 h to post-exercise, change from pre-exercise to 72 h post-exercise, change from pre-exercise to 168 h post-exercise.  4) **Muscle soreness**: self-perceived rating of muscle soreness with use of pressure algometry.  Time points measured: Immediately pre-exercise, immediately post-exercise, 24 h post-exercise, 48 h post-exercise,72 h post-exercise, 168 h post-exercise.  Analysis metric (mean ± standard deviation): Change from pre-exercise to immediately post-exercise, change from pre-exercise to 24 h to post-exercise, change from pre-exercise to 48 h to post-exercise, change from pre-exercise to 72 h post-exercise, change from pre-exercise to 168 h post-exercise.  5) **Muscle soreness**: self-perceived rating of muscle soreness while performing a bodyweight squat with use of a visual analogue scale (0 - not sore at all, 10 - extremely sore).  Time points measured: Immediately pre-exercise, immediately post-exercise, 24 h post-exercise, 48 h post-exercise,72 h post-exercise, 168 h post-exercise.  Primary, secondary, and other outcomes, including the specific measurement variable (eg, systolic blood pressure), analysis metric (eg, change from baseline, final value, time to event), method of aggregation (eg, median, proportion), and time point for each outcome. Explanation of the clinical relevance of chosen efficacy and harm outcomes is strongly recommended |
|  |  | Analysis metric (mean ± standard deviation): Change from pre-exercise to immediately post-exercise, change from pre-exercise to 24 h to post-exercise, change from pre-exercise to 48 h to post-exercise, change from pre-exercise to 72 h post-exercise, change from pre-exercise to 168 h post-exercise.  6) **Range of motion**: flexibility of the dominant exercised limb as determined by goniometry.  Time points measured: Immediately pre-exercise, immediately post-exercise, 24 h post-exercise, 48 h post-exercise,72 h post-exercise, 168 h post-exercise.  Analysis metric (mean ± standard deviation): Change from pre-exercise to immediately post-exercise, change from pre-exercise to 24 h to post-exercise, change from pre-exercise to 48 h to post-exercise, change from pre-exercise to 72 h post-exercise, change from pre-exercise to 168 h post-exercise.  7) Limb circumference: measure of leg circumference with use of standard anthropometric tape to indicate muscle swelling.  Time points measured: Immediately pre-exercise, immediately post-exercise, 24 h post-exercise, 48 h post-exercise,72 h post-exercise, 168 h post-exercise.  Analysis metric (mean ± standard deviation): Change from pre-exercise to immediately post-exercise, change from pre-exercise to 24 h to post-exercise, change from pre-exercise to 48 h to post-exercise, change from pre-exercise to 72 h post-exercise, change from pre-exercise to 168 h post-exercise. |
| Participant timeline | 13 | See Figure 2. Schedule of enrolment, interventions, and assessments. |
| Sample size | 14 | 40 participants will be recruited. The sample size is based on previous studies that compared the EIMD response between low and high load resistance exercise and reported a statistically significant between-group difference in maximal voluntary contraction change, which is deemed the most valid indirect indicator of EIMD. The mean of these values was used in a statistical power analysis, which estimated that a sample size of 8 per group is required to have 80% power to detect a difference in maximal voluntary contraction of 14% between groups, assuming a standard deviation of 10%, when using a dependent t test with a 0.05 two-sided significance level. Therefore, ten participants per group (40 total; 10 low-load male, 10 low-load female, 10 high-load male, 10 high-load female) will be recruited to allow for 20% drop-out. |
| Recruitment | 15 | Prospective participants will be invited to volunteer for the study via email and social media (Twitter, LinkedIn) advertisements, as well as word-of-mouth. |
| **Methods: Assignment of interventions (for controlled trials)** | | |
| Allocation: |  |  |
| Sequence generation, allocation concealment and implementation | 16 | Folded pieces of paper labelled either ‘LL’ (low-load) or ‘HL’ (high-load) will be placed into an opaque envelope, and one will be drawn by the participant to determine their exercise condition. To ensure an equal number of males and females in each group, separate envelopes will be used, i.e., the ‘male’ envelope will contain 10 papers labelled ‘LL’ and 10 ‘HL’, as will the ‘female’ envelope. |
| Blinding (masking) | 17 | Due to the nature of the trial (i.e., varied intensity resistance exercise) it is not possible to blind the participant nor researcher to the experimental conditions. |
| **Methods: Data collection, management, and analysis** | | |
| Data collection methods | 18a | The following assessments will be conducted in the same order during each visit for all participants:  *Blood sampling* – Blood samples will be collected from an antecubital vein of the forearm using standard venepuncture techniques into three reagent-free vacutainers (3 × 10 mL). Samples will be left at room temperature for 30 min before being stored on ice and will later be analysed for serum concentrations of creatine kinase and interleukin-6.  *Limb circumference* – Participants’ limb circumference will be measured using a standard anthropometric measuring tape at the mid, lower-, and upper- quartile points of the trochanterion-tibiale lateral site with the participant in a standing position. The mean value of the 3 sites will be used for the analysis.  *Range of motion* – Participants’ range of motion of the knee joint will be calculated as the difference between the relaxed and flexed knee joint angle, as measured using a standard goniometer with the participant in a supine position.  *Muscle soreness* – Participants will first rate their muscle soreness using a 10-point visual analogue scale ranging from ‘not sore at all’ to ‘extremely sore’ while performing a simple bodyweight squat. |
|  |  | Secondly, muscle soreness will be assessed with the pressure-pain threshold (PPT) test using a computerised pressure algometer with the participant in a supine position. The probe head (1 cm^2^) of the algometer will be placed at the mid, lower-, and upper- quartile points of the trochanterion-tibiale lateral site and increasing pressure will be applied until the participant indicated pain. The mean value of the 3 sites will be used for the analysis.  All data obtained from these assessments will be recorded on paper data collection sheets, then copied to digital format (see data management plan, 19). |
|  | 18b | Any outcome data obtained from participants who withdraw from the study will be included in the final analysis, if consent to do so is granted. |
| Data management | 19 | In order to ensure data protection, digital participant information will be stored on a password protected computer and data collection papers will be stored in a locked filing cabinet at the study site, available only to the primary investigators. Participant anonymity and confidentiality will be maintained through use of coded identification numbers, and all identifiable data will be anonymised or destroyed following analysis of the complete dataset. Anonymised data will be uploaded to the institution’s repository to be available for secondary analysis. |
| Statistical methods | 20a | Statistical analysis will be conducted using IBM SPSS (version 25, SPSS Inc., Chicago, IL). All assumptions for statistical models will be assessed using the Shapiro-Wilk and Kolmogorov-Smirnov tests, and data that violate the assumptions will be analysed using the equivalent non-parametric test. Independent t-tests/Mann-Whitney U tests with Bonferroni corrections will be used to examine any between-group differences in baseline characteristics, including body composition and habitual activity. The Levene’s test will be used to check for equality of variances between groups. A two-way mixed design analysis of variance will be used to analyse all muscle damage markers and dietary intake data between exercise conditions (low-load and high-load) and within time points (-28 d, -25 d, pre, post, +24, +48, +72, and +168 h). Data sphericity will be assessed with the Mauchly’s test and any data that violates the Greenhouse-Geisser assumptions will be corrected with Huynh-Feldt. Any significant group × time interactions will be analysed post hoc using independent t-tests with Bonferroni corrections for between-group comparisons at each time point. Within-group differences across time will be analysed using paired t-tests/Wilcoxon signed rank tests. The within-group mean change from baseline to each time-point will be reported for each muscle damage marker to allow for future calculation of effect sizes and inclusion in meta-analyses. Statistical significance will be set at P < 0.05. Confidence intervals assume 95% confidence in the range of the mean. All data will be reported as mean ± standard deviation (SD) unless otherwise stated. |
| Ethics and dissemination | | |
| Research ethics approval | 24 | This study protocol has received approval by the Department of Sport and Exercise Sciences Research Ethics Committee at Durham University (SPORT-2020-11-14T11_22_22-vlcz52; May 2021) and the Tyne and Wear South NHS Research Ethics Committee (21/NE/0073; May 2021). |
| Protocol amendments | 25 | This protocol will undergo no further amendments. |
| Consent or assent | 26a | Participants will sign a digital consent form; a link to which will be sent to them by the principal investigator. |
| Confidentiality | 27 | Participant anonymity and confidentiality will be maintained through use of coded identification numbers, and all identifiable data will be anonymised or destroyed following analysis of the complete dataset. Anonymised data will be uploaded to the institution’s repository to be available for secondary analysis. |
| Declaration of interests | 28 | The study investigators declare no competing interests, financial or otherwise. |
| Access to data | 29 | Only the study investigators will have access to the trial dataset until anonymised data is made publicly available through publication. |
| Dissemination policy | 31a | A summary of each participant’s outcome data will be sent to them individually following their completion of the trial. The complete and anonymised study data will be disseminated by publications in peer-reviewed journals and conferences. |
|  | 31c | This study protocol is intended to be made publicly accessible. |
| Appendices |  |  |
| Informed consent materials | 32 | See appendix 1. |
| **Appendix 1. Consent Form**  **Project title**: Sex differences in muscle damage following acute resistance exercise performed at high- or low- intensity  **Researcher**: Alice Pearson  **Department:** Sport and Exercise Sciences  **Contact details**: alice.g.pearson@durham.ac.uk  **Supervisor name**: Dr Karen Hind **Supervisor contact details:** karen.hind@durham.ac.uk  This form is to confirm that you understand what the purposes of the project, what is involved and that you are happy to take part. Please initial each box to indicate your agreement:   | I confirm that I have read and understand the Information Sheet dated [__/__/__] and the Privacy Notice for the above project. |  | | --- | --- | | I have had sufficient time to consider the information and ask any questions I might have, and I am satisfied with the answers I have been given. |  | | I understand who will have access to personal data provided, how the data will be stored and what will happen to the data at the end of the project. |  | | I agree to take part in the above project. |  | | I understand that my participation is entirely voluntary and that I am free to withdraw at any time without giving a reason. |  | | I understand that anonymised (i.e., not identifiable) versions of my data may be archived and shared with others for legitimate research purposes. |  | | I am not currently participating in another research project |  | | I have not participated in a research project involving dietary and/or exercise intervention in the previous 6 months |  | | To my knowledge, I am not currently pregnant nor breastfeeding (females only) |  |  | Participant’s Signature_____________________________­____ Date__________________  (NAME IN BLOCK LETTERS)_________________________________________________  Researcher’s Signature________________________________ Date__________________  (NAME IN BLOCK LETTERS)_________________________________________________ | | --- | | | |
| Biological specimens | 33 | Whole-blood samples will be centrifuged at 4°C, 4000 rpm for 15 min within 2 h of collection. Serum samples will be transferred into 1.5 mL microcentrifuge tubes and stored at -80°C until subsequent analysis. The concentrations of creatine kinase and interleukin-6 within serum will be determined with commercially available enzyme-linked immunosorbent assay kits. |

*It is strongly recommended that this checklist be read in conjunction with the SPIRIT 2013 Explanation & Elaboration for important clarification on the items. Amendments to the protocol should be tracked and dated. The SPIRIT checklist is copyrighted by the SPIRIT Group under the Creative Commons “[Attribution-NonCommercial-NoDerivs 3.0 Unported](http://www.creativecommons.org/licenses/by-nc-nd/3.0/)” license.
